# Mono-ADP-ribosylation by PARP10 inhibits Chikungunya virus nsP2 proteolytic activity and viral replication

**DOI:** 10.1007/s00018-023-04717-8

**Published:** 2023-02-25

**Authors:** Sarah Krieg, Fabian Pott, Laura Potthoff, Maud Verheirstraeten, Mareike Bütepage, Alexandra Golzmann, Barbara Lippok, Christine Goffinet, Bernhard Lüscher, Patricia Korn

**Affiliations:** 1 Institute of Biochemistry and Molecular Biology, Faculty of Medicine, RWTH Aachen University, Pauwelsstraße 30, 52074 Aachen, Germany; 2 Institute of Virology, Campus Charité Mitte, Charité—Universitätsmedizin Berlin, 10117 Berlin, Germany; 3 Berlin Institute of Health, 10117 Berlin, Germany

**Keywords:** Mono-ARTDs, ADP-ribosylation, Alphaviruses, ( +)ssRNA-viruses

## Abstract

Replication of viruses requires interaction with host cell factors and repression of innate immunity. Recent findings suggest that a subset of intracellular mono-ADP-ribosylating PARPs, which are induced by type I interferons, possess antiviral activity. Moreover, certain RNA viruses, including Chikungunya virus (CHIKV), encode mono-ADP-ribosylhydrolases. Together, this suggests a role for mono-ADP-ribosylation (MARylation) in host-virus conflicts, but the relevant substrates have not been identified. We addressed which PARP restricts CHIKV replication and identified PARP10 and PARP12. For PARP10, this restriction was dependent on catalytic activity. Replication requires processing of the non-structural polyprotein nsP1-4 by the protease located in nsP2 and the assembly of the four individual nsP1-nsP4 into a functional replication complex. PARP10 and PARP12 inhibited the production of nsP3, indicating a defect in polyprotein processing. The nsP3 protein encodes a macrodomain with de-MARylation activity, which is essential for replication. In support for MARylation affecting polyprotein processing, de-MARylation defective CHIKV replicons revealed reduced production of nsP2 and nsP3. We hypothesized that MARylation regulates the proteolytic function of nsP2. Indeed, we found that nsP2 is MARylated by PARP10 and, as a consequence, its proteolytic activity was inhibited. NsP3-dependent de-MARylation reactivated the protease. Hence, we propose that PARP10-mediated MARylation prevents polyprotein processing and consequently virus replication. Together, our findings provide a mechanistic explanation for the role of the viral MAR hydrolase in CHIKV replication.

**Supplementary Information:**

The online version contains supplementary material available at 10.1007/s00018-023-04717-8.

## Introduction

Upon viral infection, host cells initiate an antiviral immune response, which depends on various signaling processes. Post-translational modifications (PTMs) are among the quickest mechanisms to adapt to environmental changes or stressors and are thus an essential part of antiviral signaling. Viruses have developed multifaceted strategies to either evade or hijack cellular mechanisms, e.g. encoding proteins that regulate PTMs and thereby counteracting antiviral reactions of the host [1–5]. In recent years, the role of ADP-ribosylation at the host–pathogen interface became apparent [6–14]. ADP-ribosylation is a PTM of proteins, which is catalyzed intracellularly primarily by PARP enzymes (members of the ADP-ribosyltransferase diphtheria toxin-like (ARTDs) family) [1, 15]. These enzymes use NAD^+^ as co-factor to transfer ADP-ribose onto a substrate protein with release of nicotinamide. Based on their catalytic features ARTDs are subdivided into three classes. The first class contains enzymes capable of transferring multiple ADP-ribose moieties in an iterative process, thereby forming long polymers (PARP1 and 2, and TNKS1 and 2). This results in poly-ADP-ribosylation (PARylation) of substrate proteins. Enzymes restricted to mono-ADP-ribosylation (MARylation) form the second and largest class (PARP3, 4, 7, 8, 10–12, 14–17) [16–20]. The third group is defined by PARP13, which cannot bind NAD^+^ and thus appears to be catalytically inactive [21]. The discussion regarding PARP9 is controversial. It remains to be clarified whether it possesses MARylating activity in complex with DTX3L or whether DTX3L mediates the MAR transferase activity of the heterodimer [20, 22].

PARylation is best known for its function in the DNA damage response, but has also been linked to chromatin organization, ribosome biogenesis, telomere maintenance, signaling processes and cell death [1, 23, 24]. In contrast, the functions of MARylation are less well understood. It has been associated with DNA damage repair, gene expression, signaling, stress response and cell death [1, 25]. Recent findings indicate a role for MARylation in host–pathogen conflicts [1, 14, 25]. The expression of several MARylating PARPs is triggered by type I interferons (IFNs) or pathogen-associated molecular patterns (PAMPs), such as LPS, as part of an innate immune response to pathogens [6, 8, 11, 14, 26–29]. Among these PARPs are PARP10, PARP12, and PARP15, which have been identified to interfere with replication of Venezuelan Equine Encephalitis Virus (VEEV) [7, 8]. In line with this, PARP12 restricts Zika virus (ZIKV) replication [30]. In addition to these molecular and cell-based studies, evolutionary analysis suggests a role for several PARP family members, e.g. the macrodomain-containing PARPs (PARP9, 14, 15) and PARP13, at the host–pathogen interface [31]. Although catalytically inactive, the latter is best studied for restricting viral replication by recognizing foreign CG-rich RNA through its Zinc finger domains [14, 32, 33].

ADP-ribosylation can be read and regulated by macrodomains, i.e. macrodomains can function as readers or erasers of ADP-ribosylation. These structurally highly conserved protein folds are found among all domains of life and a subset of positive single-strand RNA (( +)ssRNA) viruses [14, 34–36]. While PAR chains are recognized and bound by, for example, the macrodomain of histone macroH2A1.1, the macrodomains 2 and 3 of murine Parp14 specifically interact with MARylated proteins [37, 38]. Degradation of PAR chains is mediated by PARG, which contains a macrodomain that cleaves the bond between single ADP-ribose units, but not between the amino acid and the protein proximal ADP-ribose [39, 40]. ADP-ribosylation is fully reversible because MacroD1, MacroD2 and TARG1 are macrodomain-containing hydrolases capable of removing MAR from substrates [41–43]. Besides these cellular enzymes, viral macrodomains have recently been characterized as MAR hydrolases [14]. Macrodomains found in a subset of ( +)ssRNA viruses, including members of the alphavirus genus such as Chikungunya virus (CHIKV), remove MARylation [6, 9, 10, 44]. This provides additional support for a role of MARylation in host–pathogen conflicts.

CHIKV is vector-borne and has caused epidemic outbreaks in Asia, Africa, the Americas and Europe and it is further spreading [45]. Patients suffer from an acute flu-like phase that is associated with fever, rash, and arthralgia. In addition, roughly a third of affected individuals develop chronic joint rheumatism that can last for many years [46]. Hence, this virus is a growing threat to quality of life and imposes a considerable economic burden. To date no vaccines or therapeutics have been FDA-approved, although first clinical vaccine trials are in progress and recently the first vaccine candidate finished phase III clinical trial reporting positive results (NCT04546724; https://clinicaltrials.gov/ct2/show/NCT04546724) [45, 47, 48]. Therefore, it is crucial to further elucidate the function of the non-structural proteins to identify viral or cellular therapeutic targets for CHIKV containment and treatment.

CHIKV encodes a non-structural polyprotein (nsP1234) that is translated early after infection. This protein is then cleaved by nsP2 into the 4 individual nsPs (nsP1-4) that assemble into the functional replication complex [49]. The processing occurs auto-catalytically through the protease domain of nsP2 [49]. Mutations interfering with protease activity result in defective CHIKV replication [50]. Similarly, mutations interfering with the hydrolase activity of the nsP3 macrodomain abolish replication [9, 51] (see also below). However, in contrast to nsP2, the biological function of the nsP3 macrodomain remains elusive.

Here we identified the interferon inducible PARP10 and PARP12 as host factors restricting CHIKV replication. Mechanistically, PARP10-mediated MARylation reduced the amount of processed nsPs. Similarly, the lack of a hydrolytically active macrodomain resulted in defective polyprotein processing. We identified nsP2 as a substrate for MARylation in vitro and in cells. MARylation of nsP2 by PARP10 inhibited its proteolytic activity in vitro, which supports our observation of defective polyprotein processing. We found that the MAR hydrolase activity of nsP3 removed MARylation from nsP2, thereby reactivating its proteolytic activity. Together, our data suggest a functional role for the MAR hydrolase activity of CHIKV nsP3 during viral replication and offer a mechanism how MARylation may function in host–virus conflicts.

## Results

### PARP10 and PARP12 restrict CHIKV replication

**Fig. 1:**
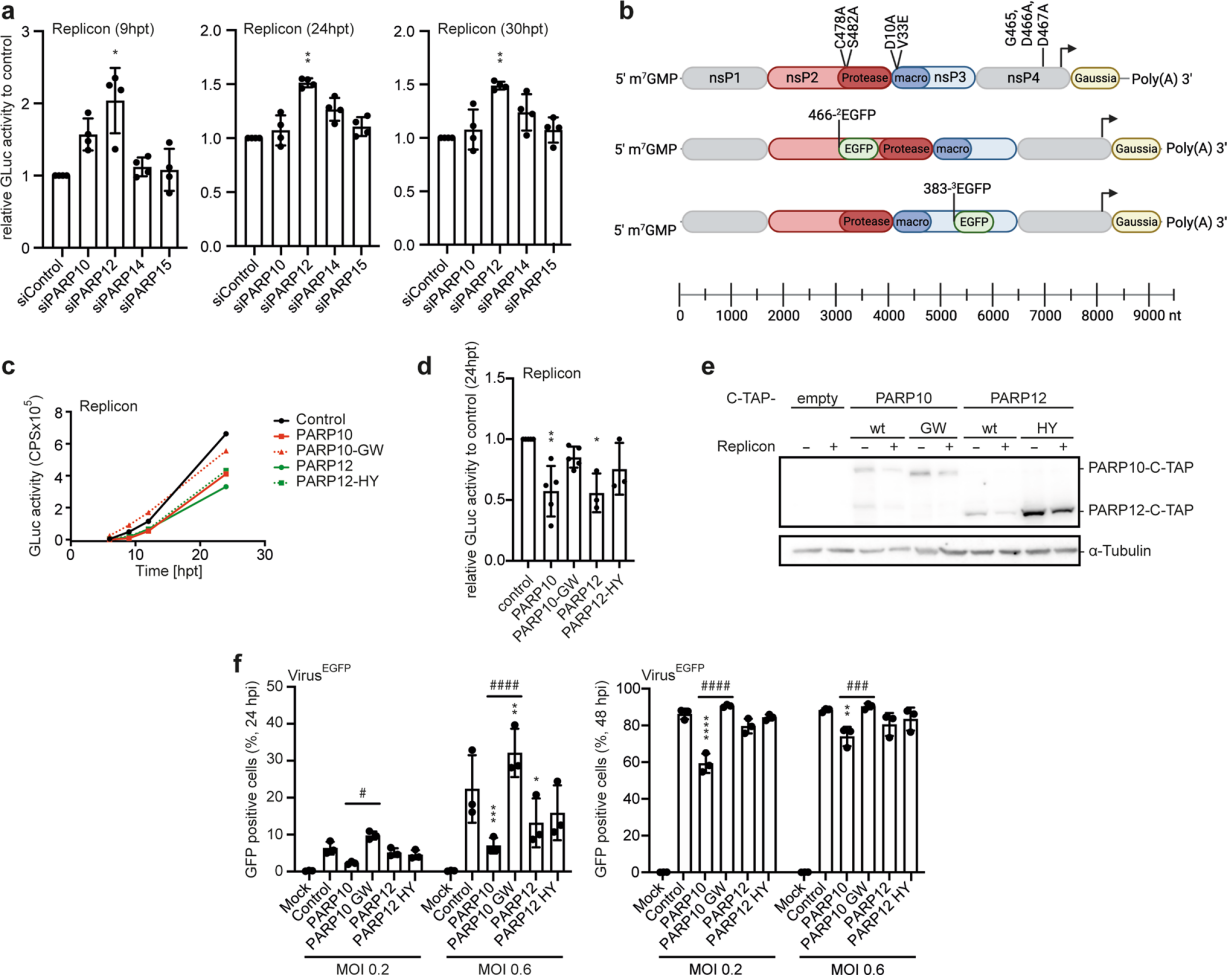
PARP10 and PARP12 interfere with CHIKV replication. (**a**) HEK293 cells were co-transfected with siRNA pools targeting the different *PARP* mRNAs as indicated and with ^3^EGFP replicon RNA. Gaussia luciferase activity was analyzed 9, 24, and 30 hpt. Normalization was against *siControl*. Error bars indicate SD (*n* = 4; 2 technical replicates measured per *n*; Kruskal–Wallis, * indicates significance compared to control). (**b**) Schematic representation of the replicons used in this study. The scale bar indicates the length of the replicon variants in nucleotides (nt) (Created with BioRender.com). (**c–e**) HEK293 Flp-In T-REx cells stably expressing TAP-tagged proteins were induced with doxycycline (Dox) 16 h prior to the transfection with replicon RNA (*n* = 3–5). (**c**) Representative determination of Gaussia luciferase activity (mean of two technical replicates). (**d**) Quantification of luciferase activity 24 h post transfection (hpt), normalized to TAP-tag control cells (control). Error bars indicate SD (*n* = 3–5; 2 technical replicates measured per *n*; Kruskal–Wallis, * indicates significance compared to control). (**e**) Whole cell lysates were analyzed for expression of PARP10 (5H11) and PARP12 (Sigma) by immunoblotting. HEK293 cells were transfected with in vitro transcribed RNA of the indicated CHIKV replicon variants (*n* = 3). (**f**) Cells were infected with fully infectious virus expressing an EGFP reporter under the control of a subgenomic promotor with the indicated MOI and analyzed for GFP expression by flow cytometry 24 (left panel) and 48 h post infection (hpi) (right panel). All error bars indicate SD (*n* = 3, 3 technical replicates were measured per *n*; two-way ANOVA, *indicates significance compared to control, #indicates significance between individual samples). (^*/#^*p* ≤ 0.05; ^**/##^*p* ≤ 0.01; ^***/###^*p* ≤ 0.001; ^****/####^*p* ≤ 0.0001)

CHIKV relies on a functional macrodomain for replication [9, 51]. This indicates strongly that the capability to bind or reverse MARylation is essential for proper virus replication. It supports the suggestion that MARylating PARPs function as antiviral host factors. To identify PARPs that affect CHIKV replication we performed knockdown experiments (Fig. 1a). HEK293 cells were transfected with siRNA oligo pools targeting the IFNα-inducible *PARP10,* *PARP12,* *PARP14,* and *PARP15* (Supplementary Fig. 1a, b and [6, 14, 52]), prior to transfection with CHIKV replicon RNA. This replicon encodes the four non-structural proteins but lacks the open reading frame for the structural proteins. Instead, the subgenomic promotor of the replicon controls the expression of Gaussia luciferase, which we analyzed as surrogate for viral replication (Fig. 1b). Because this luciferase is secreted, time course experiments are easily possible and provide an estimate of replication [53]. Luciferase was measured 9, 24 and 30 h post transfection (hpt) (Fig. 1a). At the early time point, the knockdown of both *PARP10* and *PARP12* showed an increase in replication, while the effect decreased at later time points. Knockdowns of *PARP14* or *PARP15* affected replication only mildly (Fig. 1a).

Based on these findings we decided to focus on PARP10 and PARP12. Overexpression of PARP10 and PARP12 interfered with CHIKV replication in HEK293 cells stably expressing doxycycline (Dox) inducible TAP-tagged PARP10 or PARP12, either wildtype (wt) or the catalytically inactive mutants (Fig. 1c–e, Supplementary Fig. 1c, d and [17]). Protein expression was induced by Dox prior to transfection with replicon RNA. Control cells were also treated with Dox, which had a minor effect on CHIKV replication (Supplementary Fig. 1e). PARP10 and PARP12 overexpression reduced replication of the replicon twofold, while the respective catalytically inactive variants, PARP10-G888W(GW) or PARP12-H564Y(HY), showed little effect (Fig. 1c, d). This suggested that the MARylation activity of these enzymes is required to interfere with CHIKV replication. The differences were not attributed to variations in protein expression, as the mutants were expressed more efficiently than the wt proteins (Fig. 1e, Supplementary Fig. 1d). Similarly, HEK293 cells transiently expressing PARP10 or PARP12 showed reduced replicon replication, whereas we did not observe any effect by PARP7 or PARP1, both are not induced by IFNα [14] (Supplementary Fig. 1f and g).

To expand on these findings, we determined the effects of PARP10 and PARP12 on the replication cycle of infectious CHIKV, which expresses EGFP from an additional subgenomic promoter (Fig. 1f) [54]. Overexpression of PARP10 and PARP12 decreased EGFP expressing cells as measured by flow cytometry 24 and 48 h post-infection (hpi) (Fig. 1f and Supplementary Fig. 2). In this setup, the catalytically inactive mutant of PARP12 restricted replication to the same extent as the wt protein, indicating that PARP12 may have more than one mode of action, one potentially independent of MARylation. In contrast, PARP10-GW enhanced viral replication, suggesting a dominant negative effect. This demonstrated dependency on catalytic activity of PARP10 as a CHIKV restriction factor. Of note, the inhibitory effects of PARP10 and PARP12 were more pronounced at the early time point (Fig. 1f). Taken together, MARylation driven by the IFNα responsive PARP10 and PARP12 restricts CHIKV replication.

### MARylation reduces the levels of processed nsPs

**Fig. 2:**
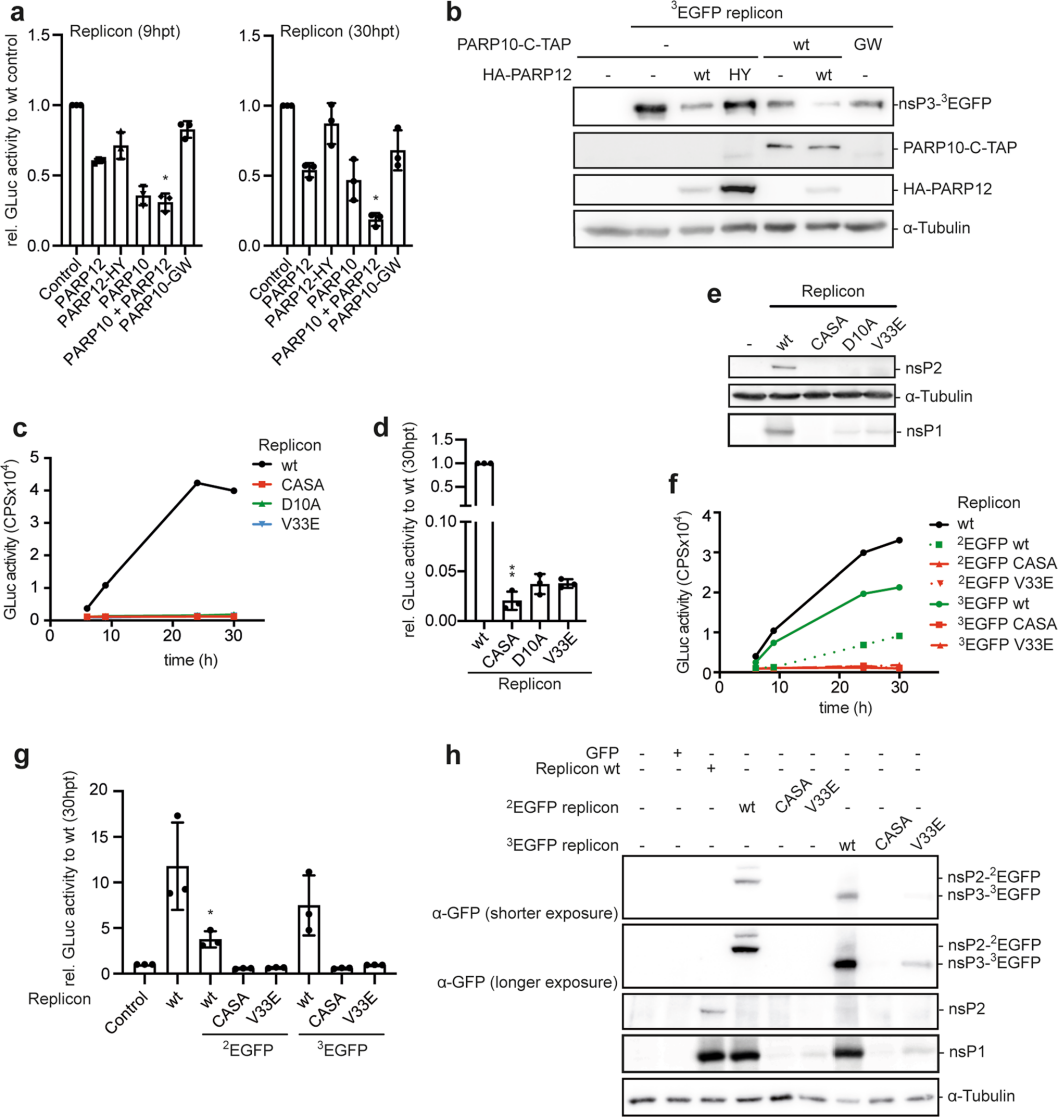
MARylation causes a reduction in processed non-structural proteins. (**a, b**) HEK293 Flp-In T-REx cells stably expressing the indicated TAP-tagged proteins were transfected with plasmids encoding HA-tagged PARP12 or PARP12-HY. Twenty-four h later, the TAP-tag proteins were induced with Dox for 16 h and subsequently transfected with ^3^EGFP replicon RNA (*n* = 3). (**a**) Gaussia luciferase activity was analyzed 9 and 24 hpt (left and right panel, respectively) and normalized to control. Error bars indicate SD (*n* = 3; 2 technical replicates measured per *n*; Kruskal–Wallis; ^*^p ≤ 0.05). (**b**) Whole cell lysates were analyzed for processed nsP3 (GFP) and expression of either PARP10 (5H11) or PARP12 (HA) by immunoblotting. (**c**) HEK293 cells were transfected with in vitro transcribed RNA of the wt replicon or mutants thereof. Representative measurements of Gaussia luciferase activity (mean of two technical replicates). (**d**) Gaussia luciferase activity was analyzed 30 h post transfection (hpt) normalized to the mean of the wildtype (wt) for each experiment. All error bars indicate SD (*n* = 3; 2 technical replicates measured per *n*; Kruskal–Wallis; ^**^p ≤ 0.01). (**e**) Whole cell lysates of the cells analyzed in panel **c** were examined for processed CHIKV nsP2 and nsP1 by immunoblotting (*n* = 3). (**f–h**) HEK293 cells were transfected with the indicated CHIKV replicon variants or EGFP as control (*n* = 3). (**f**) Representative measurement of Gaussia luciferase activity at the indicated times (mean of two technical replicates). (**g**) Quantification of several experiments as exemplified in panel e, normalized to the Replicon wt. Error bars indicate SD (*n* = 3; 2 technical replicates measured per *n*; Kruskal–Wallis; ^*^p ≤ 0.05). (**h**) Whole cell lysates were analyzed for processed nsP3 (GFP), nsP2 (GFP) and nsP1 by immunoblotting

To address possible mechanisms underlying PARP10- and PARP12-dependent inhibition of viral replication, we determined the abundance of auto-proteolytically processed nsPs. We applied EGFP-encoding variants of the replicon (^2^EGFP and ^3^EGFP, in which EGFP is integrated after amino acids 466 or 383 in nsP2 or nsP3, respectively; Fig. 1b and [55]), enabling us to visualize processed nsP2 or nsP3 proteins using a GFP-specific antibody (Fig. 2). HEK293 cells stably expressing PARP10 or PARP10-GW were transfected with or without PARP12 encoding constructs prior to transfection with ^3^EGFP replicon RNA (Fig. 2a). Analogous to our previous experiments, both PARP enzymes repressed CHIKV replication. Of note is that co-expression of PARP10 and PARP12 revealed additive repressing effects when analyzed 30 hpt (Fig. 2a). Further, we observed a reduction in processed nsP3 in the presence of either the enzymatically active PARP10 or PARP12. NsP3 was further reduced when both enzymes were expressed simultaneously (Fig. 2b; for full size blots see Supplementary Fig. 3a). These findings led us to hypothesize three scenarios of how MARylation hampers CHIKV replication. MARylation might (i) repress initial polyprotein translation, (ii) interfere with polyprotein processing, and/or (iii) decrease the stability of the viral nsPs. Any of these three effects would inhibit viral replication [30, 49]. Impaired replication of a mutant replicon with an inactive protease (nsP2-C478A/S482A, referred to as CASA) confirmed the necessity of polyprotein processing for replication, as expected (Fig. 2c, d) [50]. Similarly, a functionally active macrodomain was needed for replication as substitution of key amino acids in the macrodomain (D10A, V33E, for details of the replicon constructs see Fig. 1b) interfered with replication (Fig. 2c, d) [6, 9].

To analyze how the lack of a functional macrodomain compromised replication, we determined the abundance of proteolytically processed nsP2 (Fig. 2e, for the specificity of the antibody see Supplementary Fig. 3b and c, for full size blots see Supplementary Fig. 3d). As expected, nsP2 was detectable after transfection of the wt but not the CASA mutant replicon (Fig. 2e). Similarly, nsP2 was not detectable when expressed from hydrolase deficient replicons (Fig. 2e), again implying repression of translation, a defect in nsP2-mediated polyprotein processing, or reduced protein stability in the absence of MAR hydrolase activity. Detection of nsP1 revealed the same results, as expected (Fig. 2e). This observation was corroborated with the ^3^EGFP and ^2^EGFP replicons and mutants thereof (Fig. 2f–h). Although replication of the EGFP-encoding variants was reduced compared to the wt replicon, it remained dependent on functional protease and MAR hydrolase activities (Fig. 2f, g). As for nsP2 or nsP1, neither GFP-tagged processed nsP2 nor nsP3 were properly generated from the hydrolase deficient replicons (Fig. 2h). However, compared to the CASA mutant, at least upon longer exposure, a weak signal for processed nsP3 was detectable for the V33E mutant replicon, indicating that the loss of MAR hydrolase activity did not completely abolish polyprotein translation or processing (Fig. 2h). This was also reflected by the slightly higher replication of the hydrolase deficient mutants compared to the CASA mutant (Fig. 2d), although replication in general was strongly impaired.

**Fig. 3:**
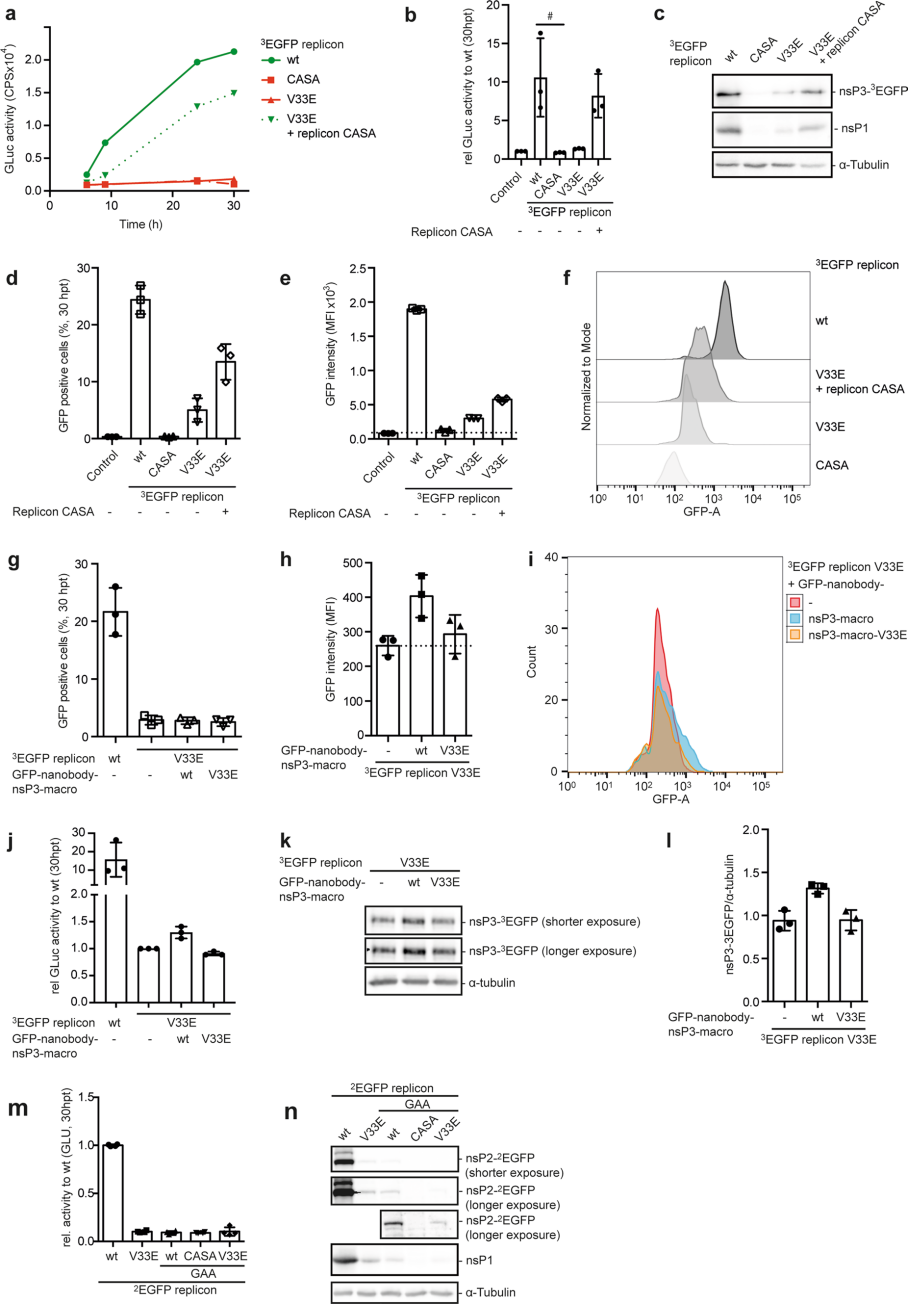
Polyprotein processing and viral replication requires both protease and MAR hydrolase activity*.* (**a–f**) HEK293 cells were (co-)transfected with RNA of the ^3^EGFP replicons and the replicon CASA mutant as indicated (*n* = 3). (**a**) Representative measurement of Gaussia luciferase activity at the indicated times (mean of two technical replicates). (**b**) Gaussia luciferase activity analyzed 30 hpt, normalized to control. Error bars indicate SD (*n* = 3; 2 technical replicates measured per *n*; Kruskal–Wallis; ^#^*p* ≤ 0.05). (**c**) Whole cell lysates were analyzed for processed nsP3 (GFP) and nsP1 by immunoblotting. (**d, e**) Flow cytometry was used to determine GFP-positive cells (**d**) as well as their mean GFP fluorescence intensity (MFI) 30 hpt (**e**) (*n* = 3). (**f**) Representative visualization of the MFI of the GFP positive cells from panels **d**, **e** with the “Modal” option scaling all channels to a percentage of the maximum count. (**g–l**) HEK293 cells were transfected with plasmids encoding anti-GFP-nanobody-nsP3-macro wt or V33E mutant. Twenty-four h later cells were transfected with in vitro transcribed ^3^EGFP wt and V33E mutant replicons as indicated. (**g, h**) Flow cytometry was used to determine GFP positive cells as well as their MFI 30 hpt (*n* = 3). (**i**) Representative visualization of the GFP intensity of the GFP positive cells. (**j**) Gaussia luciferase activity normalized to the mean of the ^3^EGFP replicon V33E for each experiment 30 hpt. Error bars indicate SD (*n* = 3; 2 technical replicates measured per n, Kruskal–Wallis). (**k, l**) Whole cell lysates were analyzed for processed nsP3 (GFP) by immunoblotting and the amount of nsP3-^3^EGFP was quantified by densitometry in relation to the loading control α-tubulin (*n* = 3). (**m, n**) HEK293 cells were transfected with in vitro transcribed RNA of ^2^EGFP replicon variants as indicated. (**m**) Thirty hpt Gaussia luficerase activity was analyzed, normalized to the ^2^EGFP wt replicon for each experiment. Error bars indicate SD (*n* = 2; 2 technical replicates measured per *n*). (**n**) Whole cell lysates were analyzed for processed nsP2 (GFP) and nsP1 by immunoblotting

ADP-ribosylation has been linked to protein degradation [56]. Therefore, we addressed whether MARylation promoted degradation of nsPs. We analyzed the amount of processed nsP2 and nsP3 and replication in presence of either the proteasome inhibitor MG-132 or the autophagy inhibitor Bafilomycin A1. These treatments were unable to rescue either the abundance of nsP2 and 3 or replication (Supplementary Fig. 4a and b), suggesting that MARylation did neither destabilize nsP2 nor 3. To separate a defect in initial translation from a processing defect, we set out to verify that polyprotein synthesis was not influenced by the individual mutations. Therefore, we performed complementation experiments (Fig. 3a–f, Supplementary Fig. 4c–h). We found that the untagged CASA replicon, containing a functional macrodomain, rescued replication of the hydrolase deficient ^3^EGFP replicon (Fig. 3a, b). Next, we analyzed GFP by immunoblotting and flow cytometry to be able to distinguish between the hydrolase deficient and the protease deficient replicons. In line with the replication rescue, an increase in processed nsP3 and nsP1 was detectable (Fig. 3c). We noted that nsP3-^3^EGFP and nsP1 were expressed from the V33E replicon, despite minimal replication (Fig. 3b, c). Moreover, considerably more cells were EGFP positive when the ^3^EGFP V33E replicon was compared with the ^3^EGFP CASA replicon (Fig. 3d), indicating initial production of the polyprotein, but inefficient and/or incomplete processing that might restrict replication. Co-transfection of the hydrolase deficient ^3^EGFP replicon with the CASA replicon resulted in a more than twofold increase in GFP positive cells, which also showed increased signal intensity, by flow cytometry (Fig. 3d–f, Supplementary Figs. 5 and 6). These findings supported the conclusion that the polyprotein was made initially, and that the hydrolase activity was important for replication.

We examined whether replication of mutant replicons (CASA or V33E) could also be rescued by co-expression of plasmid-encoded nsP2 or nsP3 (Supplementary Fig. 4c-h). Co-expression of the isolated protease domain (nsP2-459-798) partially rescued replication of the CASA mutant replicon suggesting that polyprotein synthesis took place (Supplementary Fig. 4c–e). In contrast, co-expression of nsP3 or the isolated nsP3 macrodomain was not sufficient to rescue replication of a hydrolase deficient replicon (Supplementary Fig. 4f–h). To overcome possible differences in the subcellular localization of replication hubs and the plasmid-expressed nsP3 macrodomain, we fused the macrodomain to an anti-GFP-nanobody to enhance its targeting to the replication sites of the EGFP encoding replicons. Expression of the GFP-nanobody-nsP3-macrodomain was not sufficient to enhance the number of GFP^+^ cells, which was low when transfected with the V33E mutant compared to the wt (Fig. 3g). However, we observed an increase in GFP intensity dependent on hydrolytic activity of the GFP-nanobody fusion protein by flow cytometry (Fig. 3h, i, Supplementary Figs. 5 and 6). Moreover, replication was increased (Fig. 3j), and processing of nsP3 was enhanced (Fig. 3k, l). Together, the GFP-nanobody-nsP3-macrodomain was able to rescue the hydrolase deficient ^3^EGFP replicon to some extent.

To exclude that defects in genome replication cause the decrease in amounts of processed nsPs, we manipulated the RNA-dependent RNA polymerase (RdRp) by introducing a GAA mutation into nsP4 of the ^2^EGFP replicon [55]. As expected, this resulted in a complete loss of replication (Fig. 3m). Nevertheless, we were able to detect processed nsP2 for the GAA mutant and, although less, for the GAA/V33E double mutant, confirming initial translation of the in vitro transcribed and transfected RNA (Fig. 3n). Further, the fact that the GAA/V33E double mutant replicon showed less processed nsP2 and nsP1 compared to the GAA single mutant suggests that hydrolase deficiency influences the amount of processed nsPs independent of genome replication. In line with results for the CASA single mutant, no signal for processed nsP2 or nsP1 was observed for the GAA/CASA double mutant (Fig. 3n). Taken together, these data suggest that the nsP polyprotein is initially synthesized from all replicon variants, supporting the hypothesis that MARylation affects replication at least in part by preventing polyprotein processing.

### CHIKV nsP2 is a substrate for MARylation in vitro and in cells

**Fig. 4:**
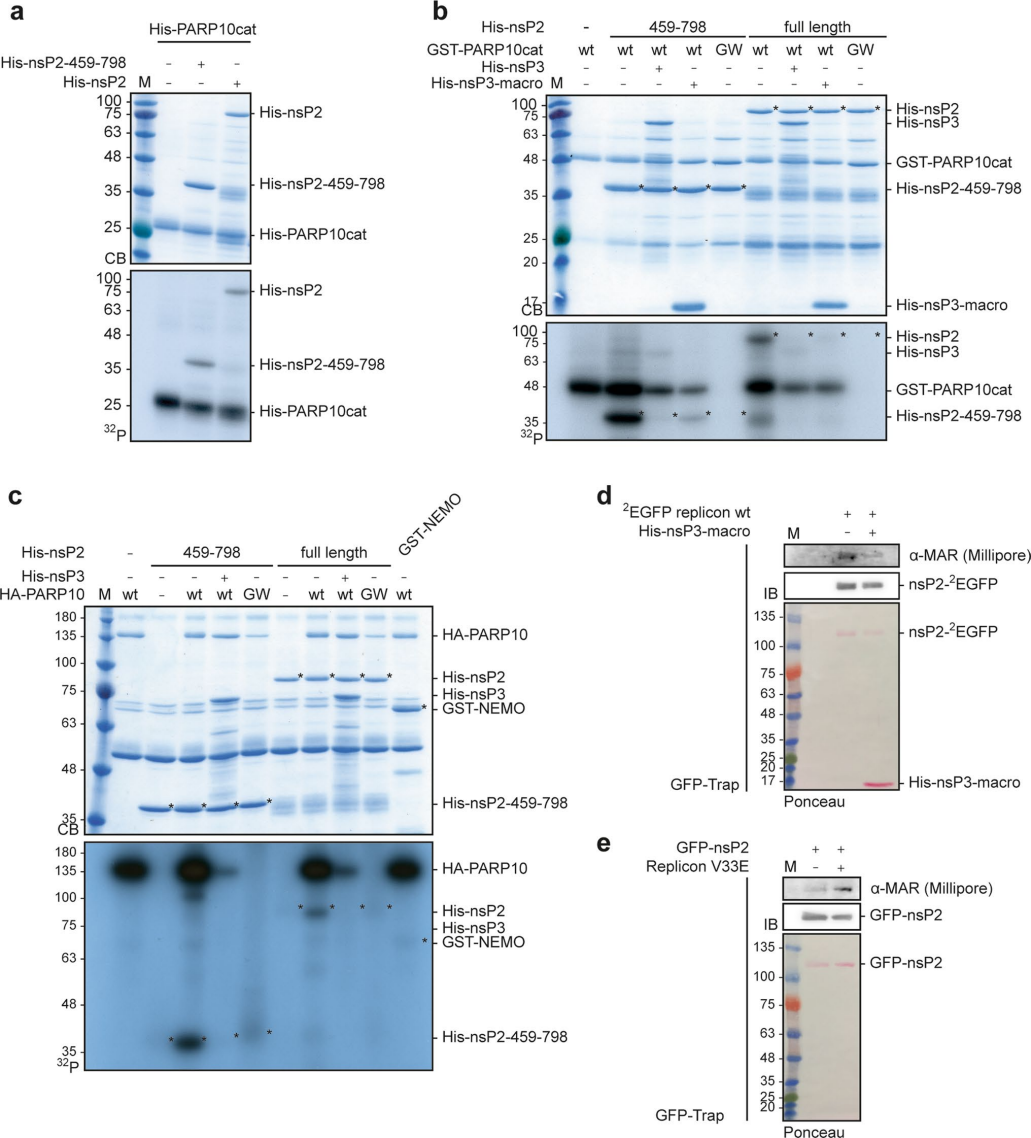
Nsp2 is MARylated by mono-ARTs and de-MARylated by the nsP3 macrodomain. (**a**) Bacterially expressed and purified His_6_-tagged PARP catalytic domains (cat) and His_6_-tagged CHIKV nsP2 or its protease domain (nsP2-459-798) were subjected to in vitro ADP-ribosylation assays using ^32^P-NAD^+^ for 30 min at 30 °C. The reactions were subjected to SDS-PAGE and the proteins were stained using Coomassie blue (CB). The incorporated radioactive label was assessed by autoradiography (^32^P) (the gel with all PARPs analyzed is shown in Fig. S4a) (*n* = 2). (**b**) Bacterially expressed and purified GST-PARP10cat and His_6_-tagged CHIKV or nsP2-459-798 were MARylated as in panel a. The catalytically inactive PARP10-G888W (GW) served as a negative control. The samples were co-incubated with His_6_-tagged nsP3 or nsP3-macro. The proteins were visualized using CB and by autoradiography (*n* = 2). (**c**) HEK293 cells were transfected with HA-tagged PARP10 or PARP10-GW, lysed and the HA fusion proteins immunoprecipitated with an HA-specific antibody. The immunoprecipitated proteins were subjected to a MARylation assay as described in panel a and b (*n* = 2). (**d**) HEK293 cells were transfected with the ^2^EGFP replicon. The cells were lysed and the EGFP fusion proteins were immunoprecipitated with GFP-TRAP-MA beads 30 hpt. The immunoprecipitates were incubated with or without His_6_-tagged nsP3-macro for 30 min at 30 °C. The proteins were analyzed by immunoblotting with a MAR-specific reagent (*n* = 1). (**e**) HEK293 cells were transfected first with plasmids encoding EGFP-nsP2, 24 h later with the V33E replicon, and 30 h later the cells were lysed. The EGFP fusion proteins were immunoprecipitated with GFP-TRAP-MA beads and analyzed by immunoblotting using a MAR-specific reagent (*n* = 1)

Consequences of protein MARylation are poorly understood. Our previous studies indicated that PARP10-dependent MARylation impairs the catalytic activity of the kinase GSK3β, which is antagonized by cellular MAR hydrolases [42, 57]. Furthermore, MARylation is reported to affect protein–protein interactions, mRNA stability and translation [1]. Following our hypothesis of a processing defect, we tested nsP2 as a substrate for MARylation. His_6_-tagged CHIKV nsP2 and nsP2-459-798, comprising the protease domain, were incubated with His_6_-tagged catalytic domains of PARP10, PARP12, PARP14 and PARP15 (Fig. 4a, Supplementary Fig. 7). The corresponding genes are IFNα responsive (Supplementary Fig. 1a and [6, 14]). Moreover, we tested PARP16, which is not regulated by IFNα (Supplementary Fig. 1a and [14]). For all catalytic domains auto-ADP-ribosylation was detected in presence of ^32^P-NAD^+^, even though the signal intensities varied considerably between the different enzymes (Fig. 4a, Supplementary Fig. 7a, b) [17, 19]. Both nsP2 and nsP2-459-798 were MARylated by the catalytic domains of IFN-regulated PARPs, but not by PARP16 (Fig. 4a, Supplementary Fig. 7a). NsP3 and the isolated macrodomain reversed PARP10 and PARP12 catalyzed MARylation of nsP2 and the protease domain (Fig. 4b, Supplementary Fig. 7b). Similarly, full length PARP10 but not PARP10-GW MARylated the protease, which was antagonized by nsP3 (Fig. 4c). As positive control GST-NEMO, which was identified earlier as substrate for PARP10, was included (Fig. 4c) [52]. To complement these in vitro findings, we measured nsP2 MARylation in HEK293 cells transfected with the ^2^EGFP replicon (Fig. 4d, Supplementary Fig. 8). The immunoprecipitated nsP2-^2^EGFP stained positive with a MAR binding reagent 30 hpt and the signal was reduced upon incubation with the recombinant nsP3 macrodomain (Fig. 4d). To analyze nsP2 MARylation over time, we immunoprecipitated nsP2-^2^EGFP 6, 9, 12, and 24 hpt. We observed a time dependent increase in nsP2 expression and a signal for MARylation of nsP2 at the latest time point (Supplementary Fig. 8a). Enrichment of nsP2-^2^EGFP after transfection of cells with either wildtype or ^2^EGFP V33E replicon RNA revealed interaction with nsP1 when using a specific CHIKV-nsP1 antibody [55] (Supplementary Fig. 8b). Further, this antibody was sensitive enough to also visualize the polyprotein. However, the polyprotein was only visible in case of the wildtype replicon and not the V33E mutant, as expected. Due to the polyprotein processing defect observed for this mutant, we argue that there is no amplification of the genome and thus no further increase in polyprotein biosynthesis beyond the initial translation of the transfected replicon RNA. Thus, the amount of polyprotein synthesized is not sufficient to be detected efficiently by immunoblotting (Supplementary Fig. 8a, b). Moreover, GFP-nsP2 MARylation was enhanced when co-transfected with the V33E replicon (Fig. 4e). Taken together, we identified CHIKV nsP2 as a new substrate for MARylation in vitro and in cells in the context of viral RNA replicon transfection.

### The proteolytic activity of nsP2 is inhibited by MARylation

**Fig. 5:**
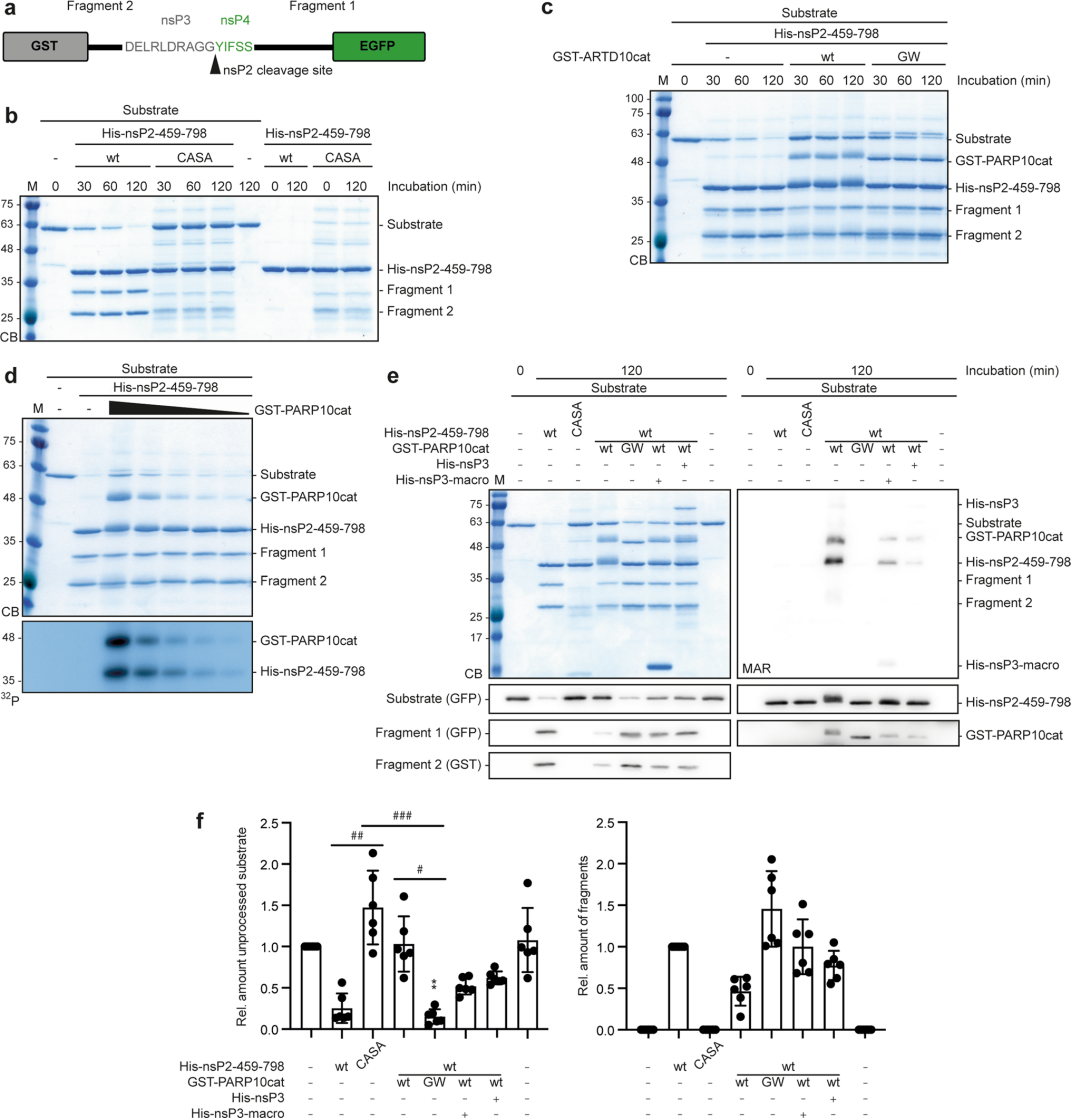
MARylation of nsP2 interferes with its protease activity. (**a**) Schematic representation of the synthetic nsP2 protease substrate with the nsP3/nsP4 cleavage site (long peptide described in [59]). (**b**) Bacterially expressed His_6_-tagged nsP2 protease domain (459–798) or the corresponding catalytically inactive CASA mutant were subjected to an in vitro protease assay with bacterially expressed and purified synthetic substrate at 30 °C for the indicated times. The reaction products were subjected to SDS-PAGE and the proteins were stained with Coomassie blue (CB) (*n* = 2). (**c**) GST-PARP10cat or the GW mutant and His_6_-tagged CHIKV nsP2-459-798 were incubated with NAD^+^ at 30 °C for 30 min. Subsequently substrate was added and further incubated for the indicated times. Proteins were analyzed by SDS-PAGE and CB staining (*n* = 3). (**d**) NsP2-459-798 was incubated with increasing amounts of GST-PARP10cat in presence of ^32^P-NAD^+^ at 30 °C for 30 min. Then substrate was added for an additional 120 min. The proteins were detected by CB and by autoradiography (^32^P) (*n* = 1). (**e**) GST-PARP10cat of the GW mutant, His_6_-tagged nsP2-459-798 or the CASA mutant, and His_6_-tagged nsP3 or nsP3-macro were incubated with NAD^+^ at 30 °C for 30 min, as indicated. Subsequently, substrate was added and further incubated for 120 min. The proteins were analyzed by CB or by immunoblotting using antibodies specific for GST, EGFP, PARP10 or nsP2 and by blotting with the MAR-specific reagent (*n* = 6). (**f**) Quantification of the experiments in panel e. The substrate (left panel) or the sum of the fragments 1 and 2 (right panel) were quantified by densitometry of the immunoblots. Error bars indicate SD (*n* = 6; Kruskal–Wallis, * indicates significance compared to unprocessed substrate, # indicates significance between individual samples, left panel). (^*/#^*p* ≤ 0.05; ^**/##^*p* ≤ 0.01; ^***/###^*p* ≤ 0.001)

Next, we aimed at determining the consequences of nsP2 MARylation on proteolytic activity. Therefore, we established a protease assay using a synthetic nsP2 substrate. The substrate consists of an nsP3/nsP4 junctional peptide (DELRLDRAGG|YIFSS) fused to GST and EGFP (Fig. 5a) [58–60]. Accessibility between the two globular tags was achieved by including a polylinker C-terminally of the cleavage site. This artificial substrate was cleaved efficiently by the recombinant nsP2 protease but not by the inactive CASA mutant (Fig. 5b). Of note, neither the C-terminal EGFP fragment (fragment 1) nor the N-terminal GST fragment (fragment 2) were further hydrolyzed, supporting the specificity of the nsP2 protease. Also, the substrate and the protease were stable when analyzed individually (Fig. 5b).

To assess the role of MARylation, we modified the nsP2 protease domain using PARP10cat. This prevented cleavage of the substrate, while co-incubation with PARP10cat-GW had no effect (Fig. 5c). Successful MARylation of nsP2-459-798 was visualized by its mobility shift on SDS-PAGE (Fig. 5c). The effect of PARP10cat catalyzed MARylation was dose dependent (Fig. 5d, Supplementary Fig. 9a). De-MARylation by nsP3 or nsP3-macro, which was evident by the reduced mobility shift or by staining with the MAR reagent, reactivated protease activity (Fig. 5e). The processing efficiency was quantified by measuring the intensities of unprocessed substrate and the two fragments by immunoblotting and densitometric scanning. This documented that MARylation by PARP10cat efficiently repressed nsP2-459-798 protease activity, which was antagonized by nsP3 or the isolated macrodomain (Fig. 5e,f). Because we noticed a weak, potential MAR signal at the size of our artificial nsP2 substrate (Fig. 5e, Supplementary Fig. 9b), we analyzed whether this potential substrate MARylation interfered with processing (Supplementary Fig. 9c). Therefore, we preincubated the substrate in presence of PARP10cat and NAD^+^ to allow modification. For control, we combined the reaction with nsP3-macro/nsP3, or used PARP10cat-GW. Then the reaction was stopped using OUL35, a selective inhibitor of PARP10 [61], before adding nsP2 to initiate processing. Preincubation and modification of the substrate did not affect its processing by nsP2 (Supplementary Fig. 9c). These findings demonstrated that MARylation inhibited reversibly with the nsP2 protease activity, while the potential MARylation of the substrate was without consequence. These findings support hypothesis that MARylation of nsP2 prevents polyprotein processing and consequently interferes with CHIKV replication. Thus, we provide a mechanism how MARylation antagonizes CHIKV replication and how the nsP3 macrodomain contributes to replication.

## Discussion

Taken together, we demonstrated that PARP10 and PARP12 interfere with CHIKV replication and identified CHIKV nsP2 as target for MARylation by IFN-inducible PARPs. Mechanistically, our results provide evidence that PARP10-dependent MARylation inhibits the nsP2 protease function, which is essential for viral replication. This results in a defect in CHIKV polyprotein processing and consequently prevents replication. This MARylation-dependent inhibition of protease activity is antagonized by the macrodomain of nsP3. Accordingly, the lack of MAR hydrolase activity hampers polyprotein processing. Thus, our findings provide evidence for a mechanism that demonstrates how MARylation can interfere with replication and provides an explanation for the importance of a functional macrodomain for the CHIKV life cycle.

PARPs have been linked to restriction of virus replication [14]. The best studied PARP family member concerning antiviral properties is PARP13 (ZAP, Zinc-finger antiviral protein). PARP13 binds viral RNA, thereby promoting its decay or blocking translation [62]. Further, PARP13 contributes to the establishment of an antiviral immune response by crosstalk with the miRNA pathway, stimulating expression of antiviral proteins, and by amplifying RIG-I signaling [62]. However, these functions are independent of ADP-ribosylation activity as PARP13 is catalytically inactive [21]. Antiviral activities have also been assigned to other PARP proteins, including PARP7, PARP10, and PARP12. Overexpression of these PARPs was shown to interfere with VEEV replication [7]. Additionally, PARP12 was described to restrict Sindbis Virus (SINV) and CHIKV replication amongst other RNA viruses [7]. PARP10, PARP12 and PARP7 have been shown to inhibit protein translation in cells infected by VEEV [8]. Our findings demonstrate that PARP10 and PARP12 interfere with CHIKV replicon replication dependent on their catalytic activities. However, replication of the full virus is also inhibited by a catalytically inactive version of PARP12 (Fig. 1f), indicating that PARP12 might possess more than one mechanism to interfere with CHIKV replication. Of note is that PARP12 and PARP13 share their domain organization [14]; thus, it is well possible that PARP12, similar to the catalytically inactive PARP13, displays antiviral activities independent of its MARylation function. PARP12 has also been identified to prevent ZIKV replication, which is mediated by depletion of the ZIKV non-structural proteins NS1 and NS3 [30]. Dependent on its catalytic activity, PARP12 has been suggested to promote PARylation of these two viral proteins. PAR chains in turn can serve as scaffold to recruit E3 ubiquitin ligases [56], and as a result modify NS1 and NS3 by K48-linked poly-ubiquitination, thereby initiating their proteasomal degradation [30]. Indeed, this concept has already been established for PARylation catalyzed by TNKS1 and 2 (tankyrase 1 and 2, or PARP5a and b, respectively) [1]. More than 70 substrates have been identified to be regulated through PAR-mediated poly-ubiquitination [56]. The proposed mechanism is that PARP12, as it is limited to MARylation, modifies NS1 and NS3, which serves as a seeding event for polymer forming PARPs, possibly TNKS1 or 2 [30]. Our findings suggest that the effect of MARylation is due to inhibition of the CHIKV protease but independent of MAR promoted nsP degradation, as inhibitors of proteasomal and lysosomal pathways did not affect nsP abundance (Fig. 3a, b). In addition to the ZIKV proteins, the nucleocapsid protein of Coronavirus (CoV) was suggested to be ADP-ribosylated during infection [63]. It will be interesting to identify the enzyme that catalyzes this modification and to define the molecular consequences.

In summary, these different reports suggest that ADP-ribosylation may interfere with multiple viral functions. This is consistent with the observation that at least six of the 12 catalytically active MARylating PARPs are induced by type I IFNs (Supplementary Fig. 1a) [1, 6, 11, 14]. The fact that the viral macrodomains display MAR hydrolase activity and thereby are able to potentially reverse MARylation of viral as well as cellular substrates supports the concept that this modification is important for the antiviral innate immune response [6, 9, 10, 14, 44]. Macrodomain mutations, which disable hydrolase activity, interfere with SINV replication in neurons and prevent neuropathy in mice [64], decrease pathogenicity and modulation of the host immune response of CoV [11, 44], and severely impair Hepatitis E virus replication [65]. Similarly, CHIKV replication is dependent on the MAR hydrolase activity of the macrodomain (Figs. 2 and 3, Supplementary Fig. 4) [9, 51]. As pointed out above, in most cases it remains to be defined what the relevant substrates of type I IFN-inducible PARPs and viral macrodomains are and how MARylation affects substrate functions. Our study provides mechanistic insight into how PARP10-mediated MARylation can interfere with CHIKV replication, in addition to the suggested translational shut off [49]. The viral macrodomain of nsP3 antagonizes the inhibition of the protease function inflicted by PARP10. Together, this links reversible MARylation of the essential viral protease to the innate immune response and to host-virus interaction.

## Materials and methods

### Cell lines and cell culture

HeLa, HEK293, HEK293 Flp-In T-REx-nsP3, -nsP3-macro, -PARP10, -PARP10-G888W [17], -PARP12, and -PARP12-H564Y cells were cultivated in DMEM supplemented with 10% heat-inactivated fetal calf serum (FCS) at 37 °C in 5% CO_2_. All HEK293 Flp-In T-REx cell lines were additionally supplemented with 15 µg/mL Blasticidin S (Invivogen) and 200 µg/mL Hygromycin B (Invivogen) for selection during every second passage. After thawing cells were regularly tested for mycoplasma by first purifying genomic DNA with the peqGOLD tissue DNA Mini Kit (peqlab) according to the manufacturer’s instructions and then a PCR reaction was performed for detection of mycoplasma DNA with specific primers (GPO-1: 5’-ACTCCTACGGGAGGCAGCAGTA-3’, MGSO: 5’-TGCACCATCTGTCACTCTGTTAACCTC-3’).

Plasmid DNA transfection of cells was performed using the calcium phosphate precipitation technique. Cells were transfected 48 h after seeding and 24 h prior to transfection with in vitro transcribed replicon RNA.

Cells were transfected with in vitro transcribed replicon RNA using Lipofectamine 2000 (Thermo Fisher Scientific) according to the manufacturer’s instructions. In short, cells were seeded in 12 well plates. For transfection 3 µg of in vitro transcribed RNA were dissolved in 100 µl OptiMEM and 5 µl of Lipofectamine 2000 were added, vortexed and incubated at room temperature for 5 min before adding dropwise to the cells. 100 µl of supernatant were collected 6, 9, 12, 24 and/or 30 h post transfection (hpt) for analysis of Gaussia luciferase activity. 30 hpt cells were used for flow cytometry analysis or lysed in RIPA buffer (10 mM Tris, pH 7.4; 150 mM NaCl; 1% NP-40; 1% DOC; 0.1% SDS; Protease inhibitor cocktail (PIC)), fractionated by SDS-PAGE and subjected to immunoblotting.

To mediate a knockdown of the gene of interest, HEK293 cells were transiently transfected with siGENOME SMARTpools (Dharmacon) directed against Non-Targeting Control #2 (D-001206-14), *PARP10* (M-014997-03), *PARP12* (M-13740-01), *PARP14* (M-023583-02), and *PARP15* (M-017186-00) using HiPerFect Transfection Reagent (QIAGEN) according to the manufacturer’s instructions for 72 h prior to transfection with in vitro transcribed replicon RNA (as described above). In short, immediately after seeding cells were transfected with a mixture of 55 µl of OptiMEM and 5 µl of HiPerFect Transfection reagent per ml of medium and a final siRNA concentration of 20 nM.

HEK293 Flp-In T-REx cells were transfected with pcDNA5/FRT/TO-N-TAP-nsP3, N-TAP-nsP3-macro, -PARP12 or the respective H564Y mutant and pOG44 (Invitrogen) using the calcium phosphate precipitation technique and selected by treating the cells with 15 µg/mL Blasticidin S (Invivogen) and 200 µg/mL Hygromycin B (Invivogen).

Sixteen h prior to transfection or infection HEK293 Flp-In T-REx cell lines were induced with 1 µg/ml doxycycline to induce the expression of stably integrated TAP-tagged constructs. Afterwards cells were transfected with in vitro transcribed replicon RNA as described above or infected with full-length virus as described below (see Virus infection and analysis of replication).

Twenty-four hpt with in vitro transcribed replicon RNA cells were treated with vehicle (DMSO), 25 µM MG132 (Sigma) or 200 nM Bafilomycin A1 (Baf.A1) (Enzo Life Sciences) for 6 h. Subsequently, supernatants were collected, and cells were lysed with RIPA buffer and subjected to SDS-PAGE and immunoblotting for analysis.

### Reagents and antibodies

The following reagents were used: β-NAD^+^ (Sigma), ^32^P-NAD^+^ (Perkin-Elmer), IFNα (Peprotech), Olaparib (Selleck Chemicals), OUL35 (Tocris) [61], propidium iodide solution (Sigma), Protease inhibitor cocktail (Sigma), Glutathione-sepharose (Sigma), TALON metal affinity resin (BD Bioscience), GFP-Trap magnetic agarose beads (Chromotek, gtma), anti-GFP (Rockland, mouse monoclonal 600-301-215 M and goat polyclonal 600-101-215), anti-α-Tubulin (Sigma, T5168 and Santa Cruz, sc-23948), anti-MAR binding reagent (Millipore, MABE1076), anti-Poly/Mono-ADP Ribose (Cell Signaling, E6F6A), anti-nsP1 (obtained from Dr. Merits, see also [55]), anti-GST (clone 6G9), anti-PARP10 (clone 5H11 [17]), anti-PARP12 (Sigma, SAB2104087), anti-Actin (clone C4, BP Biomedicals), anti-HA (BioLegend, clone 16B12), goat-anti-rabbit-HRP (Jackson Immunoresearch, 111-035-144), goat-anti-mouse-HRP (Jackson Immunoresearch, 115-036-068), goat-anti-rat-HRP (Jackson Immunoresearch, 112-035-068), rabbit-anti-goat-HRP (Santa Cruz, sc-2768).

Rabbit polyclonal, purified CHIKV-nsP2-specific antibodies were generated by immunizing rabbits simultaneously with two peptides (aa570-584: CERKYPFTKGKWNINK, and aa740-755: CVLGRKFRSSRALKPP), both located in the C-terminal third of CHIKV nsP2 (performed by Eurogentec).

### Cloning and mutagenesis

The SP6-CHIKV-replicon-SG-GLuc (hereafter referred to as replicon wt) construct was obtained from B. Kümmerer [53]. EGFP insertions were created on the basis of Utt et al. 2016 [55]. Linkers (5’-ACTAGTTCCGAGCTCGAG-3’) with restriction sites for *Spe*I and *Xho*I were introduced by PCR-based mutagenesis using the Q5 mutagensis kit (NEB) after codon 466 of nsP2 (^2^EGFP) or after codon 383 of nsP3 (^3^EGFP). The sequence encoding EGFP was amplified from pEGFP-C1 flanked by a *Spe*I restriction site and a Gly-Gly linker at the 5’-end and a Gly-Gly and a *Xho*I restriction site at the 3’-end by PCR and inserted into the linkers by restriction digestion and ligation. Single site mutations (C478A/S482A (CASA) in nsP2, D10A or V33E in nsP3 and D466A/D467A (GAA) in nsP4) were introduced into the replicon variants by insertion of custom-made DNA gBlocks (IDT). These were integrated by restriction digestion with *Nde*I for nsP2, *BstAP*I (5’-end) and *Cla*I (3’-end) for nsP3 or *Age*I (5’-end) and *Avr*II (3’-end) for nsP4 and ligation.

GST-PARP10cat constructs were described previously [17]. pDest17-PARP10cat constructs were created from pDONRZeo-PARP10cat [17] using the Gateway cloning system (Thermo Fisher Scientific). The cDNAs encoding the catalytic domains PARP12 (G480-S688), PARP14 (K1600-K1800), PARP15 (N459-A656), and PARP16 (N459-A656) were generated from plasmids obtained from H. Schüler (Stockholm) and cloned into pDest17 using Gateway cloning. pGEX4T1-PARP12cat (489-684) was created from pNIC-28-BsaI-PARP12 (M1-Q701) plasmid that was obtained from O. Gileadi (Oxford) by Gateway cloning. pDest17-nsP3, pDest17-nsP3-macro and pGEX4T1-NEMO were described previously [6, 52]. pDest17-nsP2 and pDest17-nsP2-459-798 were generated with the Gateway cloning strategy using the SP6-CHIKV-replicon-SG-GLuc as a template.

The artificial protease substrate (pGEX4T1-nsP3/nsP4-site-polylinker-EGFP) was created based on the long nsP3/nsP4 site described in Rausalu et al. [59]. This sequence was ordered as oligos containing *EcoR*I (5’-end) and *BamH*I (3’-end) restriction sites mimicking overhangs (5’- aattcGACGAGTTAAGACTAGACAGGGCAGGTGGGTATATATTCTCGTCGgag-3’, 3’-gatcctcCGACGAGAATATATACCCACCTGCCCTGTCTAGTCTTAACTCGTCg-5’) that were annealed in vitro. The sequence encoding EGFP was isolated from pEGFP-N1 using *BamH*I and *Not*I restriction sites and EGFP as well as the annealed oligos were inserted into pGEX4T1 using *Eco*RI, *BamH*I and *Not*I restriction sites and ligation. Subsequently, a polylinker was introduced into this construct for better accessibility of the protease substrate. Therefore, oligos containing this polylinker, the nsP3/nsP4 site and *EcoR*I (5’-end) and *Nco*I (3’-end) restriction site mimicking overhangs (5’- aattcGACGAGTTAAGACTAGACAGGGCAGGTGGGTATATATTCTCGTCGGAGGATCCACCGGTCGCCACCGGCTCTGCCGCTGCCACAAGAGGCTCTGCTGGAAGCGGCGGATCTGCCACAGGCTCTGGATCTGCAGCTGGCTCTGGCGACTCTGTGGCTGCCGGATCTGGCGGAGGAAGCGGCTCTAc-3’, 3’- catggTAGAGCCGCTTCCTCCGCCAGATCCGGCAGCCACAGAGTCGCCAGAGCCAGCTGCAGATCCAGAGCCTGTGGCAGATCCGCCGCTTCCAGCAGAGCCTCTTGTGGCAGCGGCAGAGCCGGTGGCGACCGGTGGATCCTCCGACGAGAATATATACCCACCTGCCCTGTCTAGTCTTAACTCGTCg-5’) were annealed in vitro and inserted into the vector using *EcoR*I and *Nco*I restriction sites and ligation.

For the anti-GFP-nanobody constructs a human optimized sequence was ordered as a custom-made DNA gBlock (IDT) containing *Age*I (5’-end) and *Xho*I(3’-end) restriction sites (5’-ACCGGTCGCCACCATGCAGGTGCAGTTGGTAGAGAGTGGGGGAGCACTTGTTCAACCTGGAGGAAGTCTGCGGCTGTCATGCGCCGCCTCAGGCTTCCCGGTGAACAGATATTCCATGCGCTGGTACCGGCAAGCACCTGGCAAGGAGAGAGAATGGGTTGCAGGAATGAGTTCCGCAGGAGACAGAAGCAGCTATGAGGATTCTGTGAAAGGAAGGTTCACTATTAGCCGGGACGATGCACGGAACACTGTGTATCTCCAGATGAATTCCCTGAAGCCGGAGGATACGGCTGTCTACTATTGTAATGTAAATGTTGGATTCGAGTACTGGGGTCAAGGAACGCAAGTGACAGTATCCAGCTCCGGACTCAGATCTCGAG-3’). This sequence was inserted into GW-pEGFP-nsP3-macro or GW-pEGFP-nsP3-macro-V33E using the *Age*I and *Xho*I restriction sites and ligation, replacing the EGFP.

pEVRFO-HA and the pEGFP-PARP10 constructs were described previously [17, 66]. pHA-, pEGFP-C1- and pcDNA5/FRT/TO-C-TAP-PARP12 were created from the pNIC-28-BsaI-PARP12 (M1-Q701) plasmid that was obtained from O. Gileadi (Oxford) by Gateway cloning. Constructs for expression of eukaryotic fusion proteins of nsP2, nsP2-459-798, nsP3 and nsP3-macro were cloned into pcDNA3-Flag, pHA, pEGFP-C1 or pcDNA5/FRT/TO-N-TAP with Gateway cloning using the SP6-CHIKV-replicon-SG-GLuc as a template. Mutants (except for replicon mutants, see above) were generated using standard mutagenesis procedures (e.g. Q5 mutagenesis kit (NEB)) and confirmed by sequencing. pcDNA3-HA-PARP1 was a kind gift from M. Hottiger (Zürich) and pCMV-HA-PARP7 from Andreas Ladurner (München).

### In vitro transcription of replicon RNA

For in vitro transcription of replicon RNA, DNA plasmids encoding the respective replicon variants were first linearized with *Nde*I. Subsequently, linearized DNA was transcribed using the mMESSAGE mMACHINE™ SP6 Transcription Kit (Thermo Fisher Scientific) according to the manufacturer’s instructions. Cap-analog [m^7^G(5')ppp(5')G] and GTP were added to the reactions to obtain 5’-capped RNA. Afterwards template DNA was digested by addition of TURBO DNase and RNA was precipitated using the lithium chloride precipitation protocol. Finally, RNA was resuspended in elution buffer from the High Pure RNA isolation Kit (Roche). Purity was controlled by agarose gel electrophoresis, concentration was measured using a NanoDrop™ 1000 (Thermo Fisher Scientific) and RNA was stored at − 80 °C until transfection.

### Purification of His_6_- and GST-tagged fusion proteins

His_6_- and GST-tagged fusion proteins were expressed in *E.* *coli* BL-21. The recombinant proteins were enriched and purified via affinity chromatography on either glutathione-sepharose for GST-fusion or TALON metal affinity resin for His_6_-fusion proteins according to standard protocols. Purification of His-nsP2-459-798, wt or inactive CASA mutant, took place without the addition of PIC to the lysis buffer.

### Replicon assays

In vitro transcribed replicon RNA was transfected into cells as described above (see’In vitro transcription of replicon RNA and Cell lines and cell culture’). 100 µl of supernatants were collected 6, 9, 12, 24 and/or 30 hpt for analysis of Gaussia luciferase activity. Cells that were not transfected with replicon RNA functioned as negative control. Gaussia luciferase is under the control of the subgenomic promoter replacing the structural proteins and secreted into the supernatant [53]. Determining the Gaussia luciferase in the supernatant can thus function as a surrogate for CHIKV replication. To analyze the luciferase activity, the BioLux® Gaussia Luciferase Assay Kit (NEB, discontinued) or the GAR-2B Gaussia Luciferase Assay (Targeting Systems) were used according to the manufacturer’s instructions following the “Stabilized Assay Protocol I”. In short, 5 ml of dilution buffer were mixed with 800 µl of stabilizer and 50 µl of 100 × substrate and incubated protected from light for 25 min at room temperature. Afterwards 5 µl of supernatant per sample were pipetted into a 96-well plate (opaque, white) in duplicates and mixed with 50 µl of substrate solution and incubated for 35–40 s. Afterwards the counts per second (CPS) were measured with a VICTOR^2^ 1420 multilabel counter (Perkin Elmer) measuring luminescence without a filter over 10 s. To determine relative replication, values were normalized to the mean value of the 2 technical replicates of the according sample and time per experiment.

### Quantitative real-time PCR

To determine ISGs among the mono-ARTs, HeLa cells were stimulated with IFNα (180 U/mL). Total RNA was isolated using the High Pure RNA isolation Kit (Roche) according to the manufacturer’s protocol. Reverse transcription was performed with 1 µg of the isolated RNA using the QuantiTect Reverse Transcription Kit (Qiagen). mRNA expression levels of *PARP3*, *PARP7*, *PARP10*, *PARP12*, *PARP14*, *PARP15* and *PARP16* were analyzed by quantitative real-time PCR (qRT-PCR) using QuantiTect Primer Assays (QIAGEN). In all settings the mRNA expression of the gene of interest was normalized to *GUS* (forward 5’-CTCATTTGGAATTTTGCCGATT-3’ and reverse 5’-CCGAGTGAAGATCCCCTTTTTA-3’; IDT).

### In vitro ADP-ribosylation assays

ADP-ribosylation assays were performed in 30 µl reaction buffer (50 mM Tris, pH 8.0, 2 mM TCEP, 4 mM MgCl_2_) with 50 µM β-NAD^+^ and 1 µCi ^32^P-NAD^+^. After 30 min incubation at 30 °C the reactions were stopped by addition of SDS sample buffer. Samples were fractionated by SDS-PAGE and gels subsequently stained with Coomassie blue to visualize the proteins. For the detection of the incorporated radioactive label, dried gels were exposed to X-ray films.

### In vitro ADP-ribosylation assays with immunoprecipitated PARP10

HEK293 cells were seeded and after 48 h transfected with plasmids encoding HA-PARP10 or the inactive GW mutant using the calcium phosphate precipitation technique. 48 hpt cells were lysed in TAP lysis buffer (50 mM Tris, pH 7.5; 150 mM NaCl; 1 mM EDTA; 10% glycerol; 1% NP-40; 2 mM TCEP; PIC) and the lysates were centrifuged at 4 °C for 30 min. HA-PARP10 was immunoprecipitated with 1 μl of anti-HA (BioLegend) antibody and protein G beads at 4 °C for 1 h. Afterwards the beads were washed in TAP lysis buffer and reaction buffer (50 mM Tris, pH 8.0, 2 mM TCEP, 4 mM MgCl_2_). ADP-ribosylation assays were carried out as described above (chapter In vitro ADP-ribosylation assays).

### In vitro protease assay

Bacterially expressed and purified His-nsP2-459-798, wt or inactive CASA mutant, were incubated with synthetic substrate in 15 µl of reaction buffer (50 mM Tris, pH 8.0, 2 mM TCEP, 4 mM MgCl_2_) for 30, 60 or 120 min at 30 °C. As a negative control substrate as well as proteases were incubated alone in reaction buffer for 0 or 120 min at 30 °C. The reactions were stopped by the addition of SDS sample buffer. Samples were fractionated by SDS-PAGE and gels subsequently stained with Coomassie blue to visualize the proteins.

### ADP-ribosylation assay with subsequent in vitro protease assay

ADP-ribosylation assays were performed in 30 µl reaction buffer (50 mM Tris, pH 8.0, 2 mM TCEP, 4 mM MgCl_2_) with 50 µM β-NAD^+^ for 30 min at 30 °C. Where indicated 10 µM of OUL35 was added to stop the ADP-ribosylation reaction. Subsequently, synthetic substrate or His-nsP2-459-798 was added to the reactions and further incubated at 30 °C for 30, 60 or 120 min. As a negative control, substrate was incubated alone in reaction buffer for 0 or 120 min at 30 °C. The reactions were stopped by the addition of SDS sample buffer. Samples were fractionated by SDS-PAGE and gels subsequently stained with Coomassie blue or subjected to immunoblotting to visualize the proteins.

### Immunoprecipitation for detection of MARylation in cells

HEK293 cells were seeded in 10 cm plates and 48 h after seeding transfected with plasmid DNA coding for GFP-nsP2 using the calcium phosphate precipitation technique or not treated. 24 h after DNA transfection or 72 h after seeding cells were transfected with in vitro transcribed replicon RNA as described above (chapter Cell lines and cell culture) but scaled up 10 × according to the amount of medium. 30 hpt cells were harvested in RIPA buffer (10 mM Tris, pH 7.4; 150 mM NaCl; 1% NP-40; 1% DOC; 0.1% SDS; PIC) and the lysates were centrifuged at 4 °C for 30 min. When immunoblotting was performed with the PAR/MAR-specific antibody, olaparib was added to the lysis buffer to prevent PARP1 activation upon cell lysis [67]. GFP-nsP2 or nsP2-^2^EGFP translated from the replicon RNA were immunoprecipitated with 5 µl GFP-Trap magnetic agarose beads (Chromotek) at 4 °C for 1 h. Afterwards beads were washed in RIPA buffer and reaction buffer (50 mM Tris, pH 8.0, 2 mM TCEP, 4 mM MgCl_2_). Subsequent hydrolase assays were carried out with bacterially expressed and purified His-nsP3-macro in 10 µl reaction buffer for 30 min at 30 °C. The reactions were stopped by the addition of SDS sample buffer. Samples were fractionated by SDS-PAGE and subjected to immunoblotting to visualize MARylation using the MAR reagent (Millipore) and the total proteins.

### Flow cytometry analysis

Thirty hpt with in vitro transcribed RNA or a plasmid encoding EGFP, cells were washed once and resuspended in 500 µl PBS containing 2% heat-inactivated FCS. For the propidium iodide (PI) single stain control, cells were then fixed and permeabilized in 80% ethanol for 30 min at -20 °C and afterwards were washed twice and resuspended in 500 µl PBS containing 2% heat-inactivated FCS. All other samples were not fixed or permeabilized. Subsequently 50 µg/ml of PI solution (Sigma) were added to all samples and incubated in the dark for 20 min. A BD FACSCanto II (BD Bioscience) was used for the flow cytometry analysis of the samples. 100,000 events were counted per sample per experiment. Evaluation of the experiments was performed with the FlowJo software (BD Bioscience).

### Virus infection and analysis of replication

Infectious CHIKV, strain LR2006-OPY, was produced by in vitro transcription of the linearized full-length viral genome including an EGFP under a second subgenomic promotor [54] and subsequent electroporation of the RNA in BHK-21 cells. The virus was passaged once in BHK-21 cells. Infections were performed under BSL-3 conditions using MOI determined by titration on HEK293T cells. Cells were fixed in 4% PFA and infection efficiency was measured as the proportion of EGFP-positive cells 24 and 48 h post infection by flow cytometry using a BD FACSLyric instrument (BD Bioscience). Evaluation of the experiments was performed with the FACSSuite v1.2.1.5657 software (BD Bioscience).

### Quantification of immunoblots and statistical analysis

Immunoblots were quantified using the Image J software (NIH, Bethesda, USA). For the statistical analysis we calculated means of technical replicates for each independent biological experiment, ending up with “n” independent datapoints (n is indicated in the figure legends). Due to variations within individual experiments (e.g. due to the quality of the invitro transcribed large replicons, transfection efficiencies, etc.), we normalized our data to 1 in several of the experiments. Thereafter, the significance was analyzed by GraphPad Prism 9.5.0 software using a nonparametric, Kruskal–Wallis test, when more than two samples were analyzed in parallel. In cases, where we used raw data as the basis of graphs and statistics, we analyzed the data by Shapiro–Wilk test, which indicated that our data likely is normal distributed. Thereafter we used two-way ANOVA. The raw and the normalized data are summarized in Supplementary Table S1.

## Supplementary Information

Below is the link to the electronic supplementary material.

Supplementary file1 (XLSX 35 KB)

Supplementary file2 (pdf 5250 KB)

## Data Availability

All data generated or analyzed during this study are included in this published article and its supplementary information files.
