# Supplementary material for: Mono-ADP-ribosylation by PARP10 inhibits Chikungunya virus nsP2 proteolytic activity and viral replication
Source: Cell Mol Life Sci. 2023 Feb 25;80(3):72. doi: 10.1007/s00018-023-04717-8 (PMC9959937; doi:10.1007/s00018-023-04717-8)
Supplement: Supplementary file 2 — Supplementary file2 (pdf 5250 KB) [file 18_2023_4717_MOESM2_ESM.pdf]

Supplementary information Krieg et al.

Mono-ADP-ribosylation by PARP10 inhibits Chikungunya virus nsP2 proteolytic activity and replication

Sarah Krieg[1], Fabian Pott[2,3], Laura Potthoff[1], Maud Verheirstraeten[1], Mareike Bütepage[1], Alexandra Golzmann[1], Barbara Lippok[1], Christine Goffinet[2,3], Bernhard Lüscher[1], Patricia Korn[1,*]

[1]Institute of Biochemistry and Molecular Biology, RWTH Aachen University, 52074 Aachen, Germany

[2]Institute of Virology, Campus Charité Mitte, Charité – Universitätsmedizin Berlin, 10117 Berlin, Germany

[3]Berlin Institute of Health, 10117 Berlin, Germany

* Corresponding author: Patricia Korn

Email: pkorn@ukaachen.de

ORCID: 0000-0002-2434-1550

Further Orcid IDs: 0000-0003-1329-0185 (Sarah Krieg), 0000-0003-3700-1691 (Fabian Pott), 0000-0001-9732-0120 (Maud Verheirstraeten), 0000-0002-3959-004X (Christine Goffinet), 0000-0002-9622-8709 (Bernhard Lüscher)

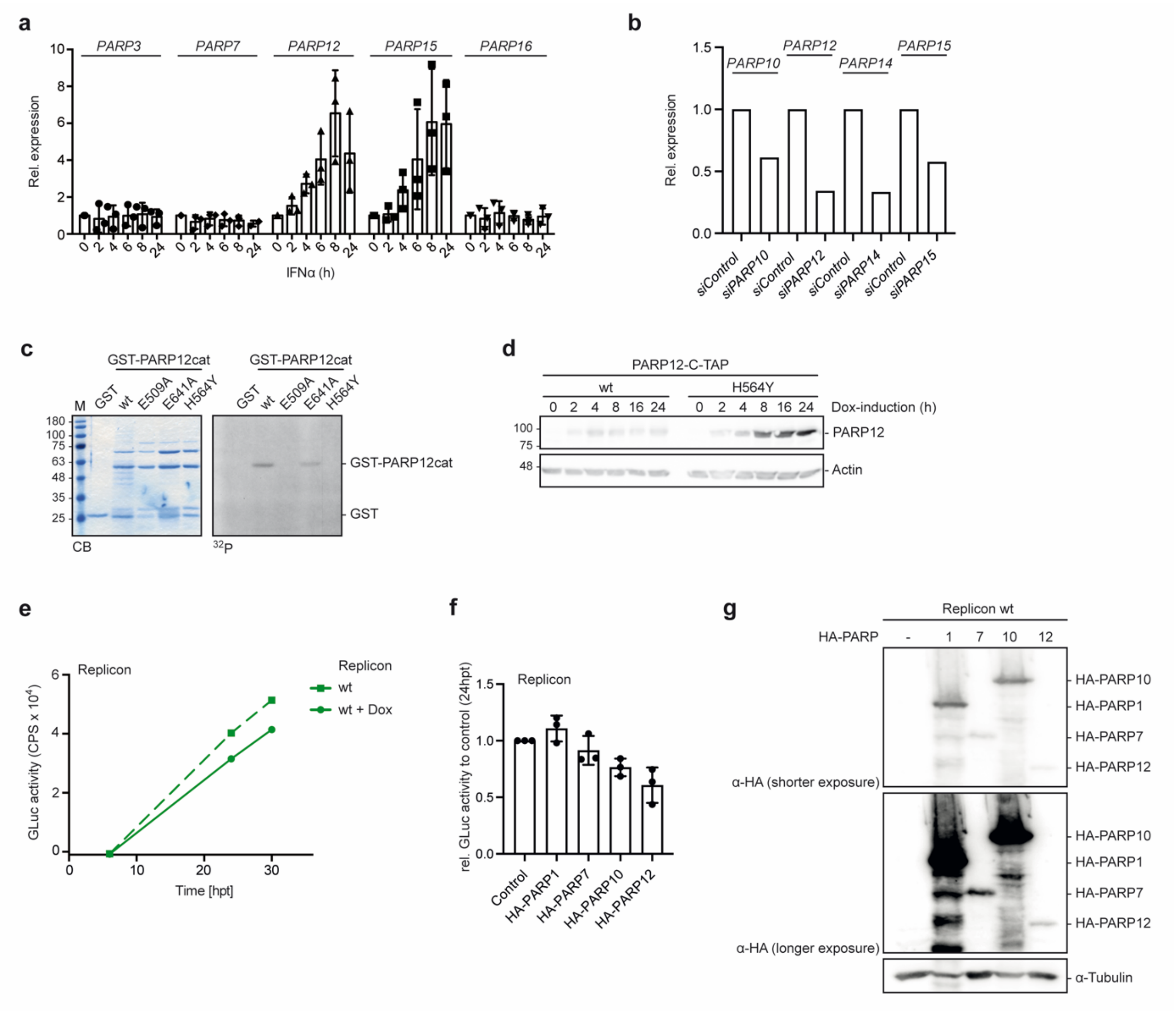

Supplementary Figure 1 (supporting Figure 1a,b-d): (a) HeLa cells were stimulated with IFNα (180 U/ml) for the indicated times. The expression of *PARP3*, *PARP7*, *PARP12*, *PARP15* and *PARP16* mRNA was analyzed using RT-qPCR and normalized to the unstimulated control. Error bars indicate SD (n = 3, 2 technical replicates were measured per n). ***(b)*** HEK293 cells were transfected with siRNA pools targeting the mRNA of *PARP10, PARP12, PARP14, and PARP15* and mRNA expression of the respective genes was analyzed by RT-qPCR 72 hpt. ***(c)*** Bacterially expressed and purified GST-PARP12cat or the indicated mutants were subjected to *in vitro* ADP-ribosylation assays in the presence of ^{32}P-NAD^{+} at 30°C for 30 min. The reactions were subjected to SDS-PAGE and the proteins were stained using CB and analyzed by autoradiography (^{32}P) (n = 1). ***(d)*** HEK293 Flp-In T-REx cells stably expressing PARP12 or PARP12-H564Y with a C-terminal TAP-tag were induced with doxycycline (Dox) for the

indicated times. Proteins were analyzed with a PARP12-specific antibody (n = 2). ***(e)*** Representative analysis of Gaussia luciferase activity in N-TAP HEK293 Flp-In T-REx cells treated with or without Dox (mean of two technical replicates). ***(f,g)*** HEK293 cells were transfected with plasmids encoding the indicated HA-tagged PARPs and 24 h later with replicon RNA (n = 3). ***(f)*** Gaussia luciferase was determined 30 hpt, normalized to the control. Error bars indicate SD (n = 3; 2 technical replicates measured per n; Kruskal-Wallis). ***(g)*** Whole cell lysates were analyzed for expression of the HA fusion proteins by immunoblotting with an HA-specific antibody 30 hpt.

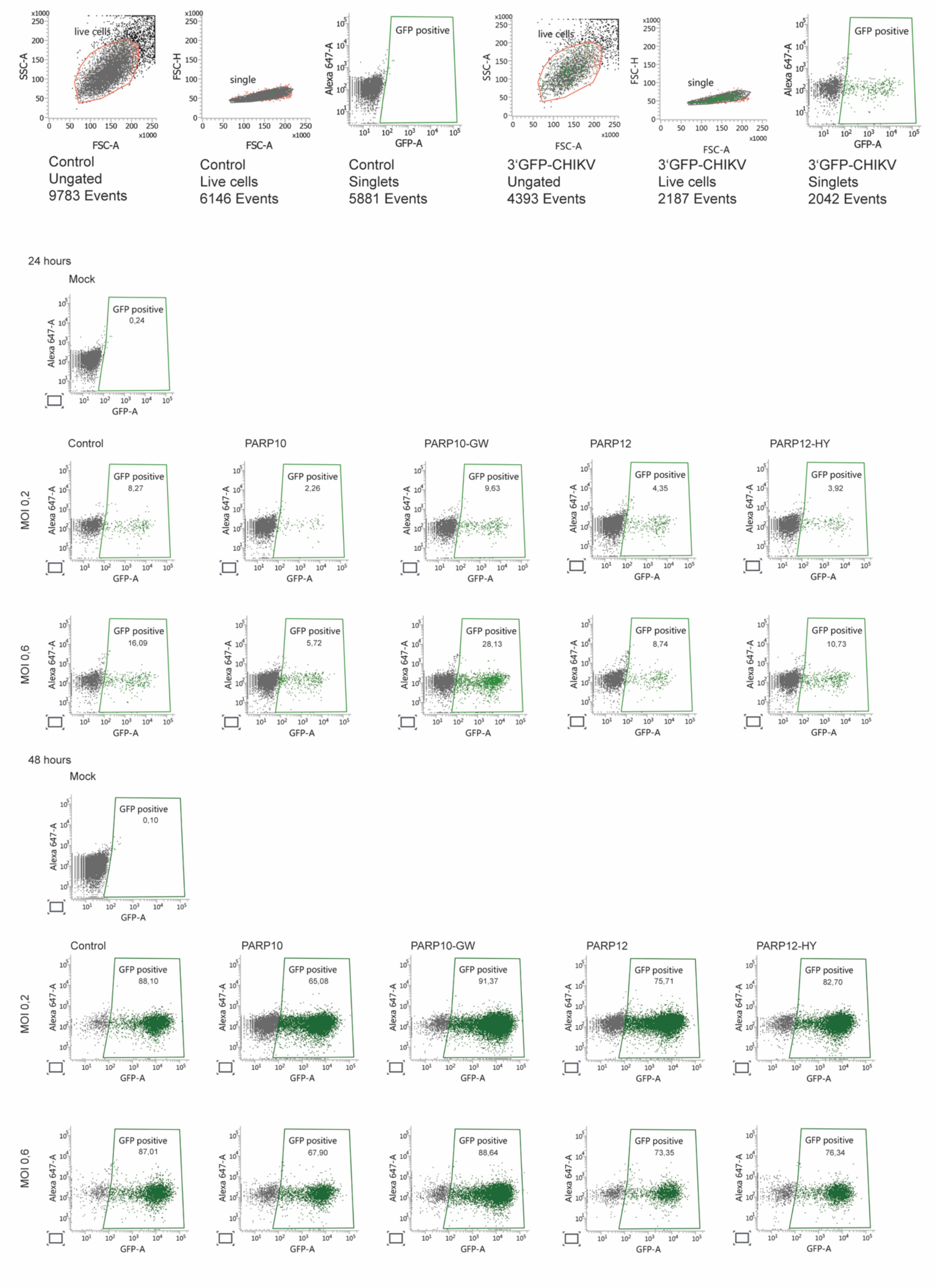

Supplementary Figure 2 (supporting Figure 1g): HEK293 Flp-In T-Rex control cells or cells infected with fully infectious virus were analyzed by flow cytometry. Depicted are representative gating procedures used to analyze all samples. First the population was gated

in SSC-A and FSC-A. Within this gate single cells were gated in the FSC-H and FSC-A. Representative gatings for each time point and both MOIs are shown and means of GFP positive cells indicated (n=3).

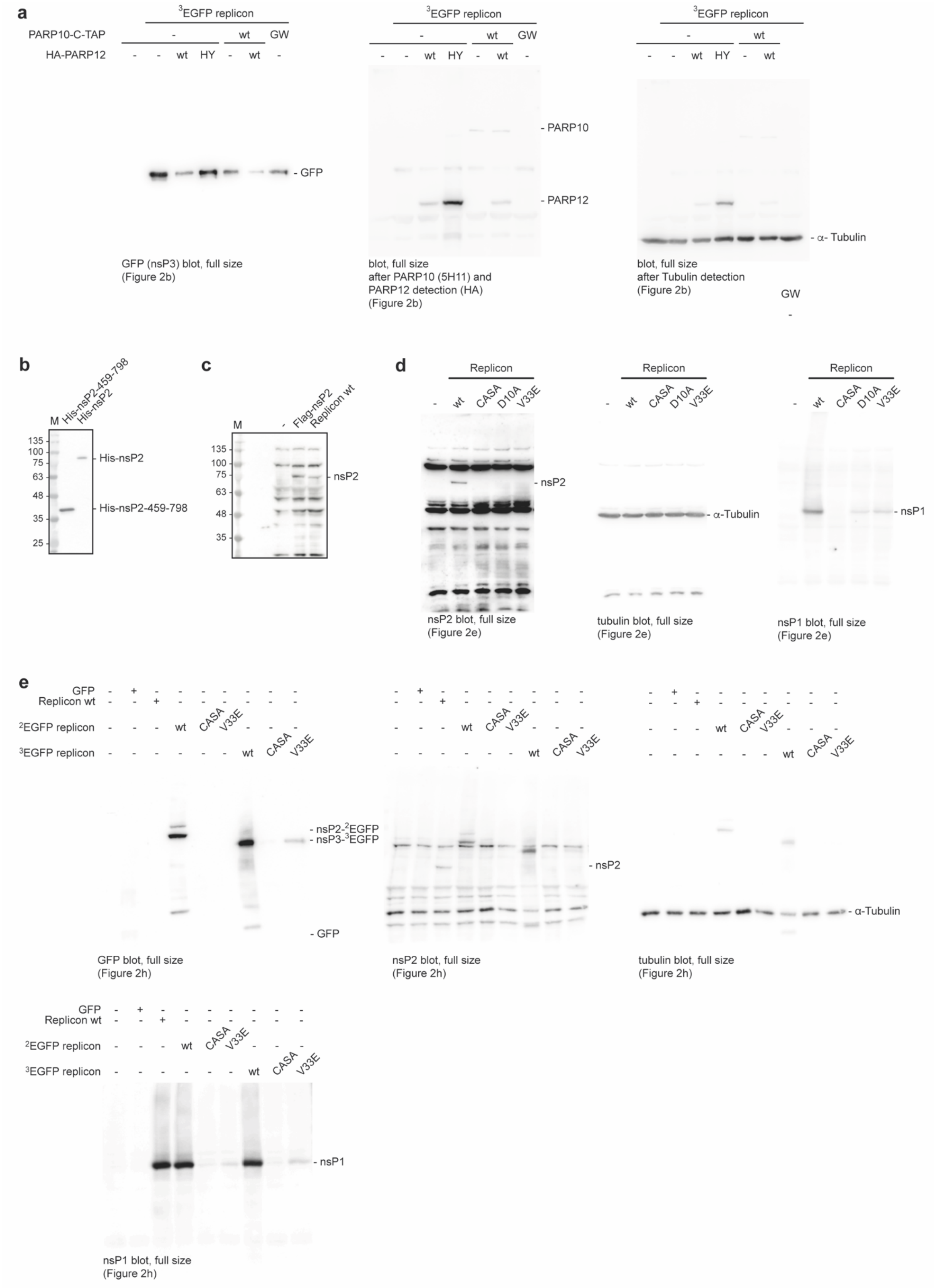

Supplementary Figure 3 (supporting Figure 2b,e, and h): ***(a)*** Shown are full size blots supporting Figure 2b. ***(b,c)*** Antibody evaluation of the custom-designed CHIKV-nsP2 antibody from Eurogentec. ***(b)*** Bacterially expressed and purified His$_6$-tagged fusions of either the

isolated protease domain (His-nsP2-459-798) or the full-length nsP2 were separated via SDS-PAGE and immunoblotted for testing the CHIKV nsP2 antibody. ***(c)*** HEK293 cells were transfected with either a plasmid encoding Flag-nsP2 or *in vitro* transcribed wt replicon RNA. Whole cell lysates from transfected cells or control cells were analyzed via immunoblotting to evaluate CHIKV nsP2 protein in these lysates using the CHIKV-nsP2 antibody. ***(d)*** Shown are full size blots supporting Figure 2e. ***(e)*** Shown are full size blots supporting Figure 2h.

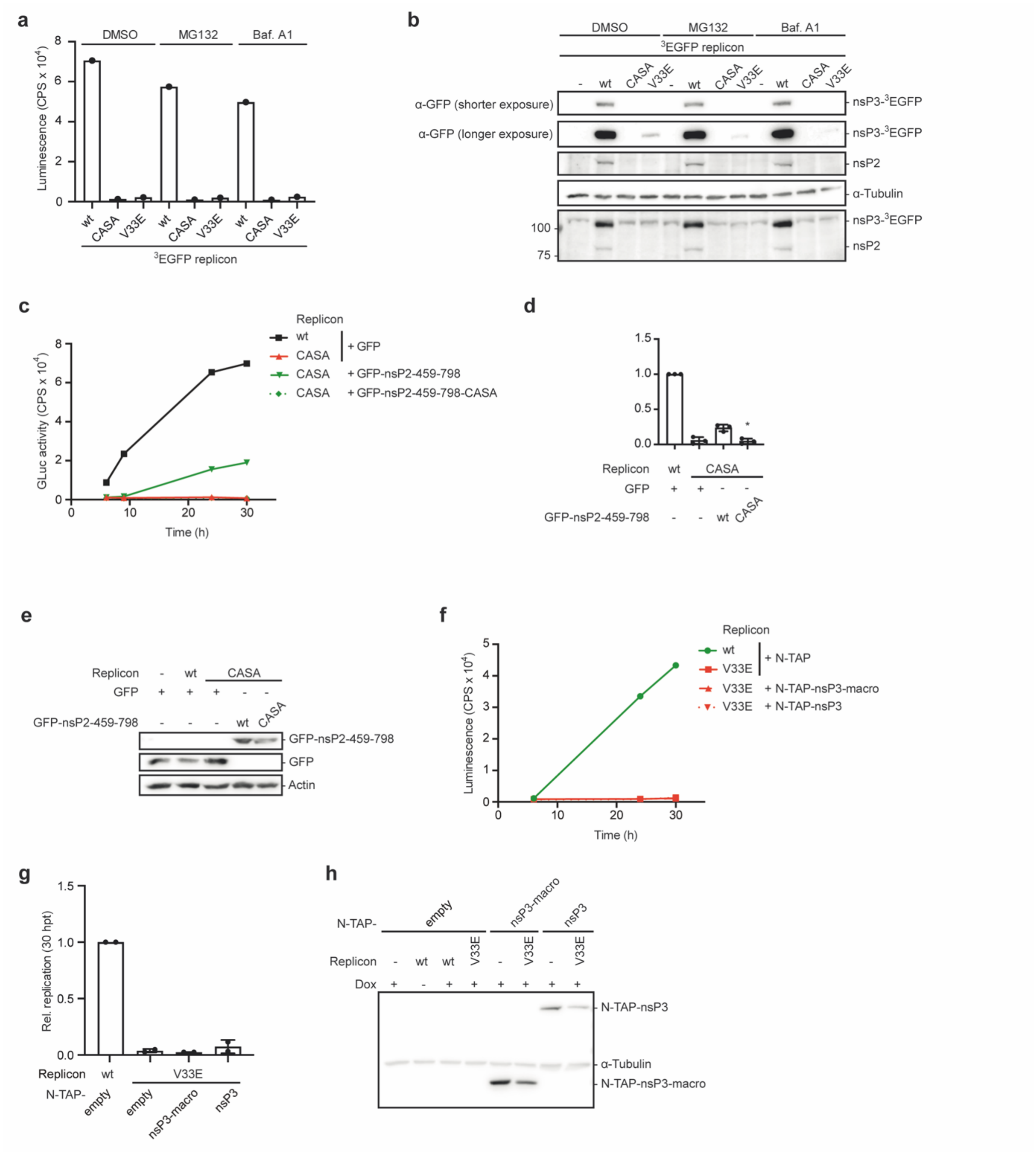

Supplementary Figure 4 (supporting Figure 3): (a,b) HEK293 cells were transfected with *in vitro* transcribed ^{3}EGFP wt and mutant replicon RNA as indicated. Twenty-four hpt, the cells were treated with either vehicle, MG132 or Bafilomycin A1 (Baf.A1) for 6 h to inhibit proteasomal or lysosomal protein degradation, respectively. (**a**) Gaussia luciferase was measured 30 hpt (n = 1; 2 technical replicates were measured). ***(b)*** Whole cell lysates were analyzed for processed nsP2 and nsP3 (GFP) by immunoblotting (n = 1). ***(c-e)*** HEK293 cells were co-transfected with *in vitro* transcribed RNA of the indicated replicon variants and

plasmids encoding either EGFP or EGFP-nsP2-459-798 fusion proteins, wt or CASA mutant, as indicated (n = 3). ***(c)*** Representative measurement of Gaussia luciferase activity (mean of two technical replicates). ***(d)*** Gaussia luciferase was determined 30 hpt, normalized to the wt replicon for each experiment. Error bars indicate SD (n = 3; 2 technical replicates measured per n; Kruskal-Wallis; asterisks * indicate significance compared to wt replicon + GFP, $^{*}p \leq 0.05$). ***(e)*** Whole cell lysates were analyzed for protein expression of the EGFP fusion proteins by immunoblotting. ***(f-h)*** HEK293 Flp-In T-REx cells stably expressing N-TAP, N-TAP-nsP3 or N-TAP-nsP3-macro were induced with doxycycline (Dox) 16 h prior to transfection with the indicated replicons (n = 2). ***(f)*** Representative determination of Gaussia luciferase activity (mean of two technical replicates). ***(g)*** Gaussia luciferase activity was measured 30 hpt. Error bars indicate SD (n = 2; 2 technical replicates measured per n). ***(h)*** Whole cell lysates were analyzed for expression of the expression of N-TAP fusion proteins by immunoblotting with an anti-rabbit secondary antibody.

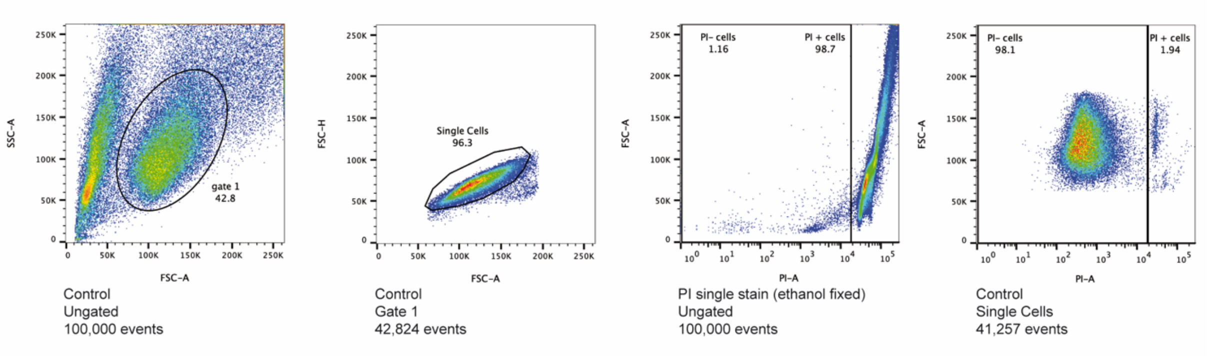

Supplementary Figure 5 (supporting Figure 3d-l): HEK293 cells were analyzed by flow cytometry with 100,000 events counted per sample per experiment. Depicted is the representative gating procedure for control cells. The same gates were applied for all samples. First the population was gated in SSC-A and FSC-A. Within this gate single cells were gated in the FSC-H and FSC-A. To gate for the propidium iodide (PI) negative single cells, control cells were fixed and permeabilized with ethanol prior to PI staining. The according gates were applied for unfixed cells as well.

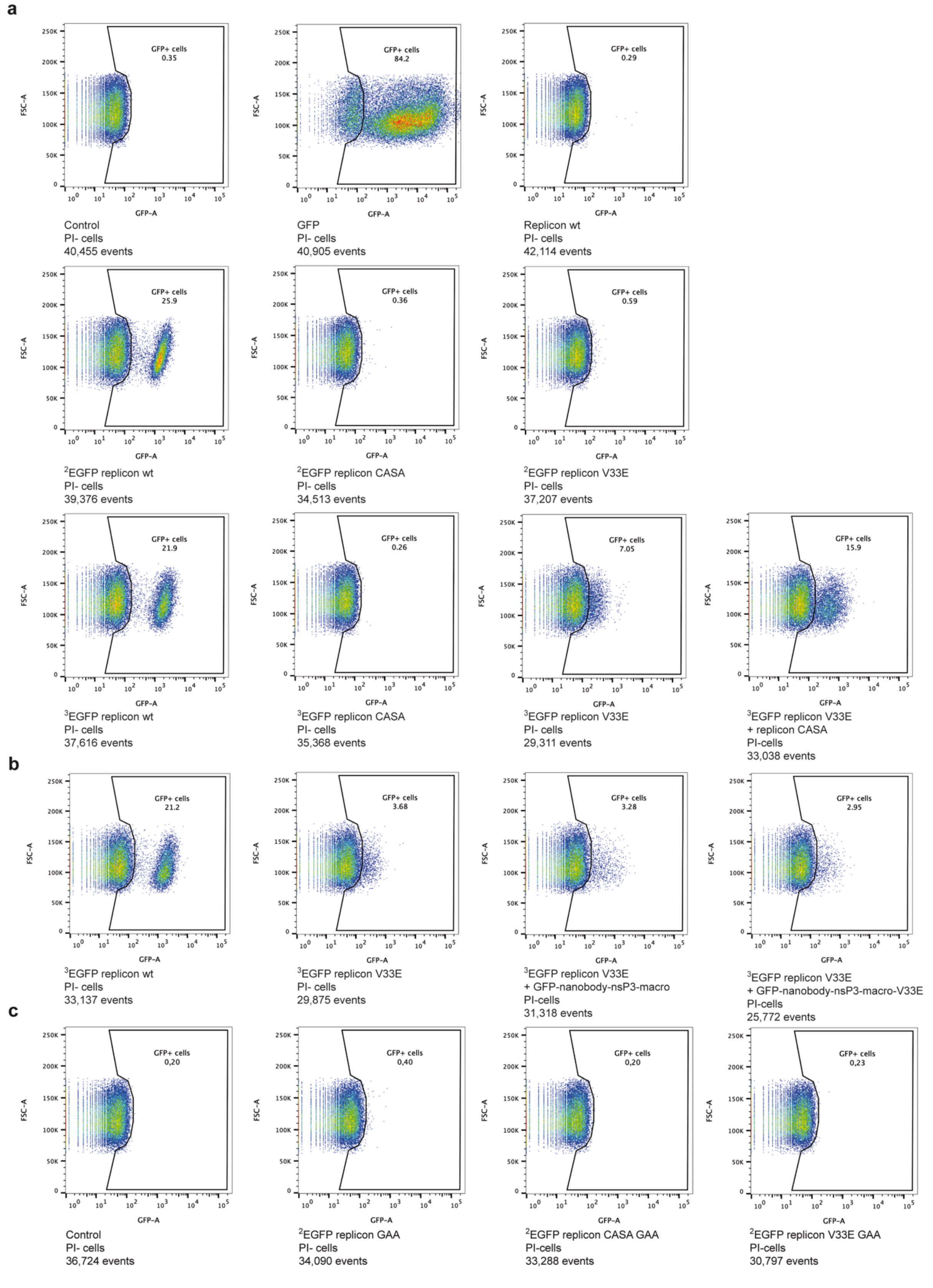

Supplementary Figure 6 (supporting figure 3d-l)*: (a-c)* Cells were analyzed by flow cytometry at 30 hpt and 100,000 events were counted per experiment (n = 3 for **a, b**; n = 2 for **c**). Depicted

are representative gatings for GFP positive cells. Within the population of PI negative, single cells (Supplementary Fig 5), a gate for GFP positive cells was set according to untransfected and EGFP transfected control cells. The gate was applied for all CHIKV replicon transfected cells and the percentage of GFP positive cells within the population is depicted. ***(a)*** HEK293 cells were transfected with the indicated *in vitro* transcribed RNA or a plasmid encoding EGFP for control (Fig. 3d-f). ***(b)*** HEK293 cells were transfected with the indicated GFP-nanobody constructs 24 h prior to transfection with *in vitro* transcribed RNA of ^{3}EGFP V33E replicon. Wt and V33E mutant replicon alone served as controls (Fig. 3g-i). ***(c)*** HEK293 cells were transfected with *in vitro* transcribed ^{2}EGFP replicon variants as indicated (Fig 3m-l).

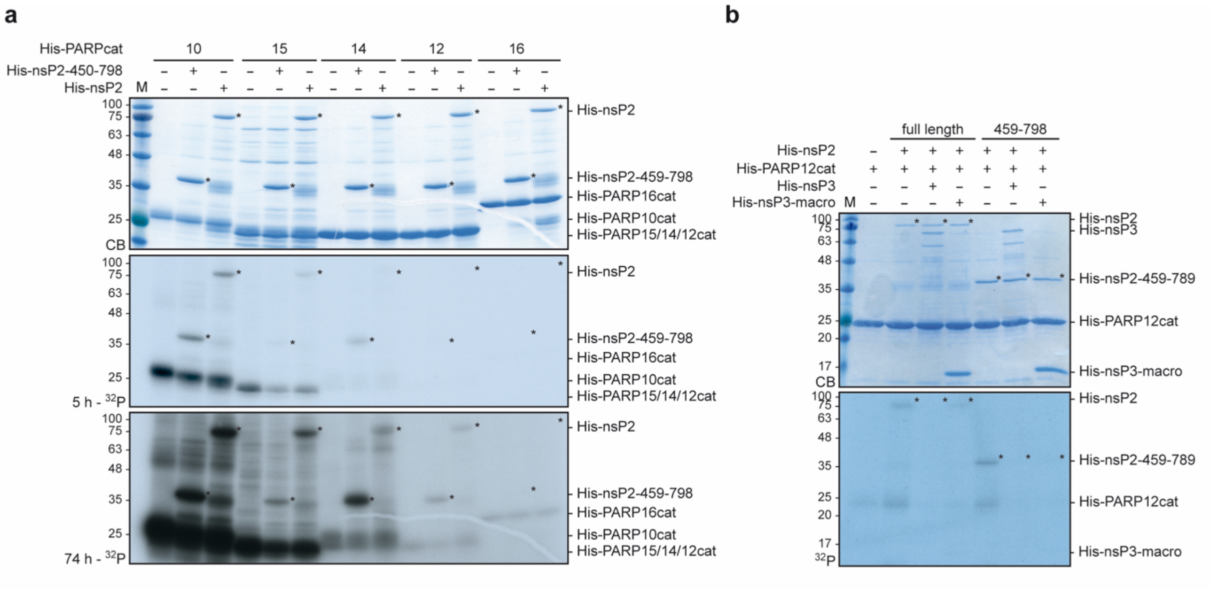

Supplementary Figure 7 (supporting Figure 4): ***(a)*** Bacterially expressed and purified His_6-tagged PARPcat domains and His_6-tagged nsP2 or nsP2-459-798 were used in *in vitro* ADP-ribosylation assays with ^{32}P-NAD$^+$ at 30°C for 30 min. The proteins were subjected to SDS-PAGE and stained using Coomassie blue (CB). The incorporated radioactive label was assessed by autoradiography (^{32}P) (n = 2). ***(b)*** Bacterially expressed and purified His_6-tagged PARP12cat and His_6-tagged nsP2 or nsP2-459-798 were used and analyzed as in panel a. In addition, His_6-tagged nsP3 or nsP3-macro were co-incubated as indicated (n = 2).

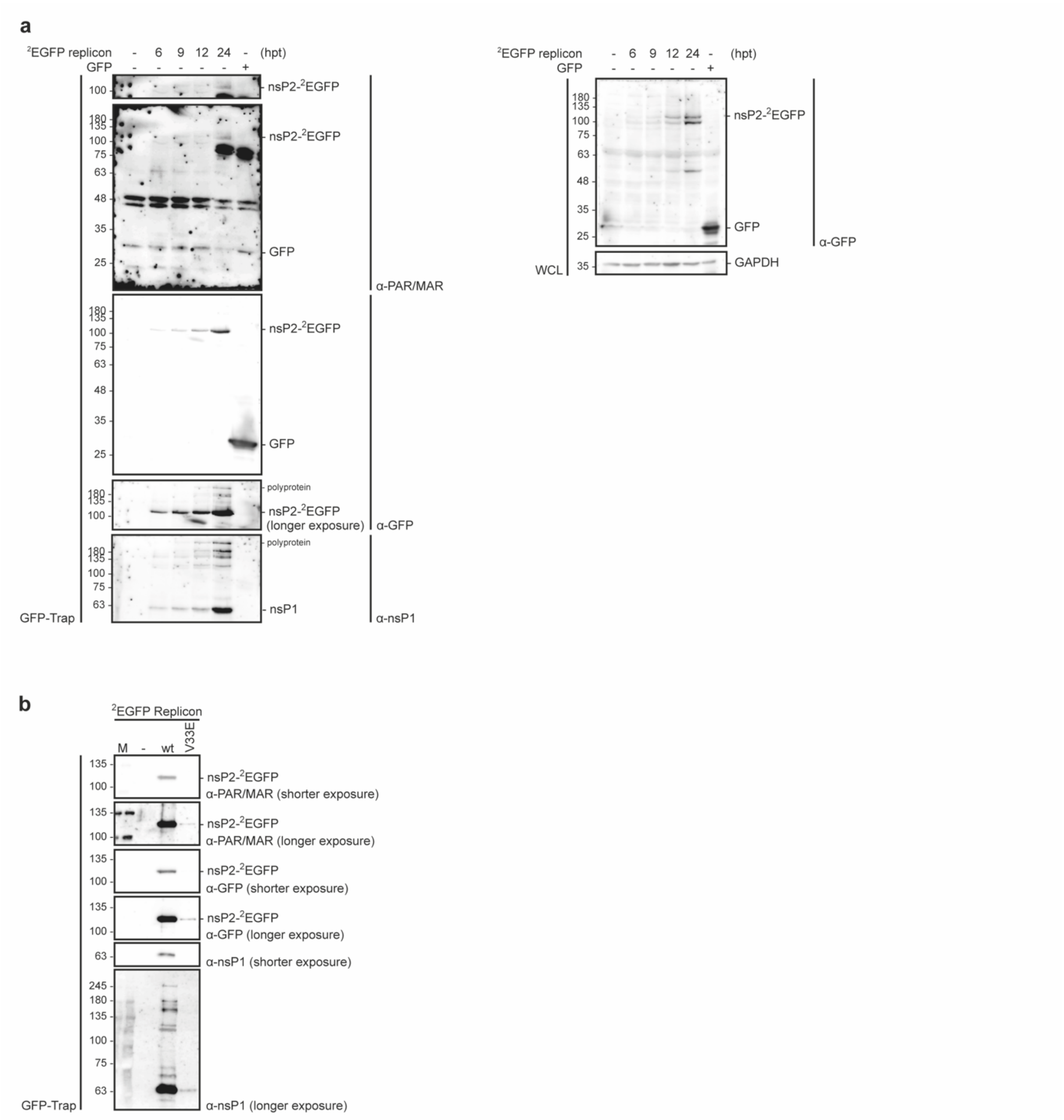

Supplementary Figure 8 (supporting Figure 4d,e): (a) HEK293 cells were transfected with *in vitro* transcribed ^{2}EGFP replicon RNA. Cells were lysed at the indicated times post transfection and nsP2-^{2}EGFP enriched using GFP-TRAP-MA beads. Immunoprecipitated material and whole cell lysates were analyzed by immunoblotting using the indicated, specific antibodies. ***(b)*** HEK293 cells were transfected with *in vitro* transcribed ^{2}EGFP replicon RNA, wt or V33E. Cells were lysed and nsP2-^{2}EGFP enriched using GFP-TRAP-MA beads. Immunoprecipitated material was analyzed by immunoblotting using the indicated antibodies.

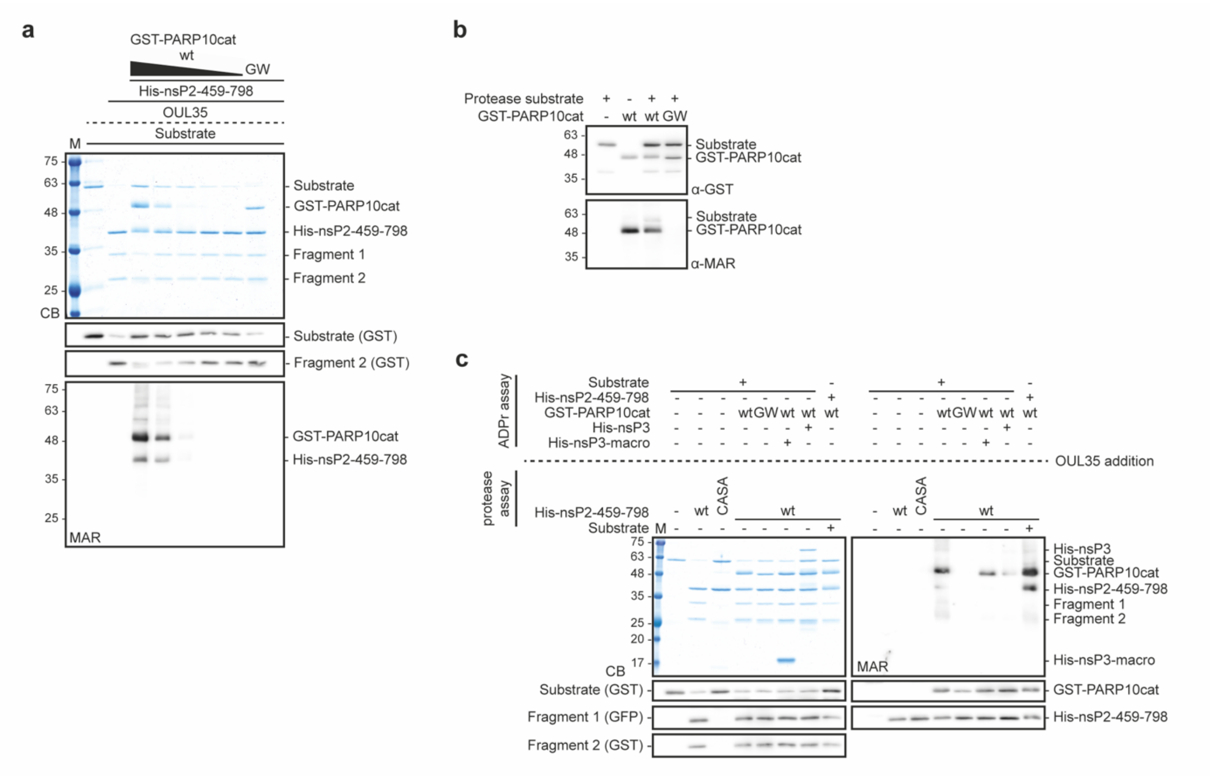

Supplementary Figure 9 (supporting Figure 5c-f): ***(a)*** His-nsP2-459-798 was incubated with increasing amounts of GST-PARP10cat or with GST-PARP10cat-GW for control in presence of β-NAD^+ at 30°C for 30min. The ADP-ribosylation reaction was stopped by addition of 10 µM OUL35 before substrate was added and cleavage allowed to occur at 30°C for additional 120 min. Proteins were separated by SDS-PAGE and analyzed by Coomassie-blue staining and immunoblotting using the indicated specific antibodies (n = 2). ***(b)*** GST-PARP10cat or its catalytically inactive mutant (GW) were incubated with the protease substrate in presence of β-NAD^+ at 30°C for 30 min. Proteins were analyzed by immunoblotting using a specific PAR/MAR-antibody to determine potential substrate modification (n = 1). ***(c)*** Substrate or His-nsP2-459+798 were preincubated in presence of GST-PARP10cat and β-NAD^+ at 30°C for 30 min as indicated and the ADP-ribosylation reaction was stopped by addition of 10µM OUL35. Subsequently, His-nsP2-459-798, its catalytically inactive mutant (CASA), or the substrate were added as indicated to allow processing at 30°C for additional 120 min. Proteins were

separated by SDS-PAGE and analyzed by Coomassie-blue staining and Immunoblotting using the indicated specific antibodies (n = 3).

Statistical information, exact P-values:

(***P-values for Figure 1, panel a***: *siControl vs. siPARP12 9h P = 0.0159; siControl vs. siPARP12 24 h P = 0.0013; siControl vs. siPARP12 30h P = 0.0073;* ***for panel d***: *control vs. PARP10 P = 0.0083; control vs. PARP12 P = 0.0155;* ***for panel f***: *(left panel) PARP10 vs PARP10-GW P = 0.0475 (MOI 0.2); control vs. PARP10 P = 0.0003 (MOI 0.6); control vs. PARP10-GW P = 0.0094; control vs. PARP12 P = 0.0139; PARP10 vs PARP10-GW P = < 0.0001 (MOI 0.6); (right panel) control vs. PARP10 P = < 0.0001 (MOI 0.2); PARP10 vs. PARP10-GW P = < 0.0001 (MOI 0.2); control vs PARP10 = 0.001 (MOI 0.6); PARP10 vs. PARP10-GW P = 0.0003 (MOI 0.6)).*

(***P-values for Figure 2, panel a:*** *9 hpt: control vs. PARP10 + PARP12 P = 0.0148; 30 hpt: control vs. PARP10 + PARP12 P = 0.0193;* ***panel d***: *wt vs. CASA P = 0.0091;* ***panel g***: *wt vs. ^{2}EGFP wt P = 0.0277).*

(***P-values for figure 3, panel b***: *wt vs. CASA P = 0.0250).*

(***P-values for Figure 5, panel f***: *(left panel) substrate alone vs. GST-PARP10-GW P = 0.0035; His-nsP2-459-798 alone wt vs. CASA P = 0.001; His-nsP2-459-798-CASA vs. GST-PARP10cat-GW P = 0.0002; GST-PARP10cat-wt vs. GW P = 0.0215).*

(***P-values for Supplementary Figure 4, panel d***: *wt + GFP vs. CASA + GFP-nsP2-459-798 CASA P = 0.0372).*
